# Leveraging prompt-driven generative AI for systematic reviews in digital psychiatry: A stage-matched comparative proof-of-concept for healthcare researchers and clinicians

**DOI:** 10.1371/journal.pdig.0001666

**Published:** 2026-09-10

**Authors:** Karisa Parkington, Jithin T. Joseph, Katie Musleh, Marianne Rouleau-Tang, Annabelle Persaud, Alice Rueda, Bazen Gashaw Teferra, Huda F. Al-Shamali, Wanying Mao, Fatemeh Gholamali Nezhad, Lisa Burback, Yanbo Zhang, Rakesh Jetly, Eric Vermetten, Candice Monson, Sri Krishnan, Richard J. Zeifman, Andrew Greenshaw, Wendy Lou, Venkat Bhat

**Affiliations:** 1 AI for Mental Health Program (formerly Interventional Psychiatry Program), Department of Psychiatry, St. Michael’s Hospital, Unity Health Toronto, Toronto, Ontario, Canada; 2 Department of Psychiatry, Temerty Faculty of Medicine, University of Toronto, Toronto, Ontario, Canada; 3 Department of Psychiatry, Faculty of Medicine and Dentistry, University of Alberta, Edmonton, Alberta, Canada; 4 Institute of Mental Health Research, The Royal Ottawa Mental Health Centre, Ottawa, Ontario, Canada; 5 Department of Psychiatry, Faculty of Medicine, University of Ottawa, Ottawa, Ontario, Canada; 6 Department of Psychiatry, Leiden University Medical Center, Leiden, The Netherlands; 7 Department of Psychology, NYU Grossman School of Medicine, New York, United States of America; 8 Department of Psychology, Toronto Metropolitan University, Toronto, Ontario, Canada; 9 Department of Electrical, Computer, and Biomedical Engineering, Toronto Metropolitan University, Toronto, Ontario, Canada; 10 Department of Brain Sciences, Faculty of Medicine, Imperial College London, London, United Kingdom; 11 Division of Biostatistics, Dalla Lana School of Public Health, University of Toronto, Toronto, Ontario, Canada; Durham University, UNITED KINGDOM OF GREAT BRITAIN AND NORTHERN IRELAND

## Abstract

The scale and pace of evidence generation in digital psychiatry increasingly exceed the capacity of traditional systematic literature review (SLR) methods. Large language models (LLMs) are gaining traction in evidence synthesis, yet limited guidance exists for integrating generative AI into transparent, reproducible SLR workflows. To develop and evaluate a prompt-driven, modular decision-making framework for adaptable SLR workflows in digital psychiatry, and to compare stage-matched performance relative to a consensus-based human reference process. We conducted a mixed-methods, stage-matched comparative evaluation of three GPT-5 mode variants (Auto, Agent, and Deep Research) against a consensus-based human-led SLR workflow using a registered digital psychiatry review (PROSPERO CRD42025648122) as a case example. Nine SLR tasks were implemented within the ChatGPT interface using structured RISEN prompts: preliminary searches; research question, eligibility criteria, and search strategy development; article screening; data extraction; article summarization; critical appraisals; and descriptive results synthesis. Performance was evaluated using task-specific assessments of accuracy, sensitivity, specificity, inter-rater agreement, reporting compliance, hallucination monitoring, and workflow feasibility. All GPT-5 mode variants were feasible across SLR stages, although performance varied by task and mode. No fabricated study-level content was identified during structured hallucination auditing. Auto mode performed best for structured rule-based tasks requiring efficiency and implementation-ready outputs (e.g., eligibility criteria). Agent mode excelled in conceptual integration and interpretive tasks (e.g., research question generation, critical appraisals, descriptive synthesis). Deep Research mode most closely approximated human reasoning for higher-order synthesis tasks, performing best in full-text article screening, article summarization, and narrative synthesis. Prompt-driven LLM workflows are feasible and semi-efficacious for selected SLR tasks in digital psychiatry when deployed within a modular, human-in-the-loop decision-support framework. Findings support task-specific deployment of an adaptable modular framework, enabling healthcare researchers and clinicians to modify LLM workflows according to methodological appropriateness, confidence in performance, and institutional review practices.

## Author summary

Systematic literature reviews (SLRs) are essential for summarizing scientific evidence, but they are often time-intensive and require substantial human effort. Recent advances in artificial intelligence (AI), including large language models (LLMs) such as ChatGPT, have raised interest in whether these tools could support or accelerate parts of the review process. However, little is known about how well different AI configurations perform across the many stages of a systematic review. In this proof-of-concept study, we evaluated an adaptable, step-by-step workflow showing how healthcare researchers and clinicians could practically use different versions of GPT-5 to assist with nine major stages of an SLR in psychiatry. Rather than testing a fully automated system, we examined how AI might support specific parts of the process while keeping human researchers involved in reviewing and verifying outputs. We found that performance varied depending on both the task and GPT-5 mode used. Some modes performed well for structured tasks such as screening and summarizing findings, whereas all modes demonstrated limitations in extracting numerical data and critically evaluating evidence quality. Overall, our findings suggest that AI may meaningfully support evidence synthesis for end-users with limited AI background when carefully supervised, but currently works best as a flexible decision-support tool rather than a replacement for expert judgement.

## Introduction

Systematic literature reviews (SLRs) are foundational to evidence-based medicine [1,2], informing clinical guidelines, healthcare decision-making, and policy development. However, the exponential growth of biomedical, psychiatry, and digital health literature has increasingly challenged the feasibility of conducting timely, comprehensive evidence syntheses [3,4], with publication volume now exceeding the practical capacity of human reviewers [5–7]. Traditional SLRs rely heavily on labour-intensive manual processes and often require more than a year to complete [8–11], increasing the likelihood that findings become outdated by publication [12,13] and limiting the responsiveness of clinical recommendations to rapidly evolving evidence.

This challenge is particularly relevant in psychiatry and health sciences research, areas undergoing rapid transformations through the integration of artificial intelligence (AI), machine learning, and digital health technologies. Contemporary AI-driven psychiatric research increasingly incorporates multimodal data streams, such as electronic health records, ecological momentary assessments, neuroimaging, wearable biometrics, smartphone sensing, and digital phenotyping, to support precision psychiatry approaches aimed at improving individualized diagnosis, symptom monitoring, treatment selection, relapse prediction, and longitudinal disease modelling [14–18]. These approaches are contributing to increasingly personalized and data-intensive models of mental healthcare while simultaneously accelerating the pace, scale, and complexity of psychiatric evidence generation. Intervention platforms, predictive models, and monitoring technologies evolve rapidly, and evidence bases expand faster than conventional review workflows can accommodate [13,17]. Consequently, the growing volume and heterogeneity of psychiatric evidence further intensifies the need for scalable, reproducible, and methodologically rigorous evidence synthesis workflows capable of supporting timely clinical translation and more responsive guideline development.

Notably, traditional human-led systematic reviews are themselves imperfect processes. Even under duplicate-review and consensus-based workflows, trained human reviewers demonstrate measurable rates of omission and misclassification during screening and data extraction tasks [12,19]. Empirical evidence suggests that abstract-screening decisions in medical systematic reviews may achieve approximately 80% - 90% accuracy under standard practice conditions, with residual disagreement and reviewer error rates ranging from 6% - 20% depending on the review context and clinical domain [19]. Human reviewers are also susceptible to fatigue, interpretive drift, anchoring effects, and variability in judgement across complex or ambiguous studies [20], challenges that may be exacerbated by pressures for speed and keeping pace with rapidly evolving evidence. This growing mismatch between the volume of published research and the capacity of human reviewers threatens the ability of evidence-based medicine to remain current, comprehensive, and clinically actionable.In response, comparative evaluation of AI-assisted SLR procedures may represent a promising approach to support more timely, scalable, and methodologically rigorous evidence synthesis, particularly within rapidly evolving domains such as psychiatry and digital health.

AI-driven tools have emerged as a promising strategy to reduce the time and cognitive load associated with evidence synthesis [21]. Recent systems (e.g., Rayyan, Elicit, Review Copilot, GPT-5) have demonstrated measurable efficiency gains across review development, screening, data extraction, and summarisation tasks [22,23] (see S1 Text). In particular, large language models (LLMs) present a potentially transformative alternative: a single, general-purpose AI system capable of executing multiple interdependent stages of the SLR pipeline using prompts and instructions in ordinary language rather than code [24–26]. Despite these advances, research in LLM workflows for SLRs is preliminary: current AI tools typically only focus on isolated tasks (most often citation screening or preliminary data extraction) rather than multi-task pipelines and often lack direct comparison with human researchers [27–29]. Furthermore, many AI tools require sophisticated configuration or application programming interface (API) access (limiting access for healthcare researchers and clinicians with limited technological expertise), depend on proprietary architectures with limited reproducibility, and lack transparent benchmarking against human reviewers [30,31]. Notably, much of the existing literature has focused primarily on isolated benchmarking tasks or engineering-focused evaluations conducted within computational research environments (see S1 Text for a review of existing literature and evidence across SLR tasks). An additional important consideration is that comparisons between LLM-assisted and human-led workflows should be interpreted relative to current best-practice evidence synthesis processes, rather than against an assumed infallible reference standard, given the known susceptibility of human reviewers to omission, interpretive variability, and judgement-related biases.

At the same time, a growing body of work highlights important limitations and failure modes of LLM-assisted SLR workflows. A recent scoping review concluded that, although applications now span most review stages, fully validated, production-ready implementations remain rare, with performance highly context-dependent [28,29]. Title and abstract screening studies similarly report that LLMs can miss eligible studies, with sensitivity estimates varying substantially across prompts, datasets, and implementation strategies (~ 0.46 – 0.98 across published studies), often accompanied by unstable specificity trade-offs and residual omission risk, thereby necessitating robust human oversight, rather than fully autonomous deployment [27,28,32–34]. Likewise, AI-assisted systematic review workflows are known to introduce distinct sources of error and bias. LLM outputs may be influenced by prompt phrasing, incomplete user instructions, context-window limitations, probabilistic reasoning patterns, and/or variability in source retrieval, potentially contributing to omission, misclassification, extraction inaccuracies, or inconsistent methodological interpretation across review stages [22,28,29]. Methodological commentaries further caution that apparent gains in data extraction accuracy may not generalize beyond the validation set and that current evaluation designs often underestimate misclassification, bias, and variability across review topics [35]. In addition, analyses of LLM-generated references for systematic reviews demonstrate high hallucination rates and low precision, particularly for GPT-class models used in scientific writing, underscoring the need for systematic verification of citations and bibliographic outputs [36].Consistent with emerging frameworks for trustworthy AI implementation (e.g., National Institute of Standards and Technology [NIST] AI Risk Management Framework [37]), AI-assisted evidence synthesis workflows may benefit from structured human oversight to identify, verify, and manage risks associated with omission, misclassification, unsupported outputs, and downstream error propagation during scientific information processing.

Despite these advances, comparatively limited attention has been devoted to operationalizing reproducible, prompt-driven workflows that can be realistically implemented by healthcare researchers and clinicians using widely accessible conversational interfaces. Consequently, despite rapidly increasing interest in AI-assisted evidence synthesis, there remains limited guidance regarding how non-technical health science researchers can systematically integrate LLMs into real-world review workflows using transparent and adaptable prompting procedures. Accordingly, both human-led and AI-assisted evidence synthesis workflows should be conceptualized as inherently fallible, probabilistic systems that require structured oversight, verification, and transparent reporting procedures to support methodological rigour, reproducibility, and trustworthy scientific interpretation.

Although GPT-class models are among the first LLMs to demonstrate efficiency and effectiveness across all stages of the SLR process [32,38–47] (see S1 Text), limited evidence supports the use of a single LLM across the core tasks of an SLR pipeline, with benchmarked performance against a human-conducted review using identical eligibility criteria and source materials. Furthermore, ChatGPT’s user interface allows users to provide task descriptions and commands in ordinary language, rather than code, drastically improving the versatility and accessibility of LLM tools for health sciences researchers and clinicians with limited technical knowledge. These natural language capabilities make ChatGPT an ideal tool to leverage for SLR prompt-driven workflow pipelines in healthcare.

To address these gaps, we conducted a clinically oriented stage-matched comparison of a nine-task prompt-driven SLR workflow specifically designed to be adaptable for implementation in a conversational interface (ChatGPT) by healthcare researchers and clinicians. Using a structured prompting framework mimicking human decision-making to enhance consistency and reduce variability (RISEN), we evaluated how three GPT-5 mode variants completed nine tasks essential to the SLR workflow, benchmarked to the performance of trained human reviewers. Importantly, the present study was intentionally designed as a clinically oriented, proof-of-concept comparative evaluation of a prompt-driven AI-assisted systematic review workflow, rather than as a large-scale benchmarking study across multiple evidence domains. Our objective was to evaluate whether a structured, operationally feasible workflow implemented through a widely accessible conversational interface could support healthcare researchers and clinicians across the major stages of evidence synthesis without requiring advanced programming expertise or custom engineering pipelines. Consistent with current best practices in evidence synthesis, the present workflow was not intended to represent a fully autonomous end-to-end systematic review system. Rather, the study was designed as a modular decision-support framework in which healthcare researchers and clinicians may selectively implement some or all task-specific components according to methodological appropriateness, confidence in LLM performance, and existing institutional workflows (e.g., librarian-supported searching, researcher-led screening, or human verification procedures). Evaluating all major downstream SLR tasks within a single study was intended to provide practical guidance regarding where prompt-driven LLM assistance may be most effectively deployed and where sustained human oversight remains essential. To facilitate reproducibility and practical translation, all RISEN-based prompts used throughout the workflow are provided. These prompts were not merely illustrative examples but empirically evaluated workflow tools benchmarked against a human consensus-reference process. By providing structured prompts that can be directly adapted for use in ChatGPT or other generative AI platforms, the present study aims to support more reproducible and standardized implementation of AI-assisted synthesis workflows within digital psychiatry while lowering technical barriers for researchers and clinicians without advanced AI expertise.

Thus, our objectives were to: (1) develop a prompt-driven workflow for key stages of the SLR process that could be adapted for digital psychiatry reviews; (2) evaluate the feasibility, accuracy, sensitivity, specificity, inter-rater agreement, reporting compliance, and hallucination monitoring of LLM-assisted execution of the SLR workflow using three GPT-5 mode variants (Auto, Agent, and Deep Research) relative to a consensus-based human workflow; (3) assess the quality, completeness, and internal consistency of LLM outputs at each review stage; and (4) systematically document task-specific errors and hallucinations to characterise mode-dependent failure patterns within the SLR process. By providing a direct, stage-matched comparison between AI-assisted and consensus-based human SLR workflows, empirically characterizing mode-dependent performance and failure profiles across the SLR workflow, and releasing a reusable structured prompting framework for immediate implementation, this study advances not only methodological understanding of LLM-assisted evidence synthesis, but also the practical translation of AI-assisted review workflows into applied healthcare research settings.

## Methods

### Study design

We conducted a parallel, stage-matched comparative evaluation of three mode variants of an LLM-assisted SLR workflow relative to the traditional human-led SLR process, following the Data, Evaluation, Algorithm, Labels, and Bias (DEAL-B) framework (S1 File). This study was intentionally not designed to evaluate a fully autonomous start-to-finish SLR pipeline. Rather, we examined GPT-5 performance across nine key SLR tasks with emerging evidence in favour of generative AI deployment (summarized in S1 Text) as part of a modular decision-support framework intended to inform selective implementation of AI assistance within real-world healthcare evidence synthesis workflows. In this way, the study aimed to identify where LLM support may be appropriate, where mode-specific strengths and limitations emerge, and where sustained human oversight remains necessary. Importantly, this study does not evaluate a fully autonomous start-to-finish SLR pipeline, as we first need to understand LLM performance at each task within the same pipeline. Instead, we examine here LLM performance across all constituent stages of the review process under structured human oversight. A pre-registered SLR with meta-analysis examining immersive virtual reality (VR) exergaming interventions for depression (PROSPERO CRD42025648122) was used as a case example. VR interventions represent an expanding area of clinical and behavioural research, with a diverse and heterogeneous evidence base, making this a stringent test case for evaluating multi-stage LLM performance.

**Fig 1:**
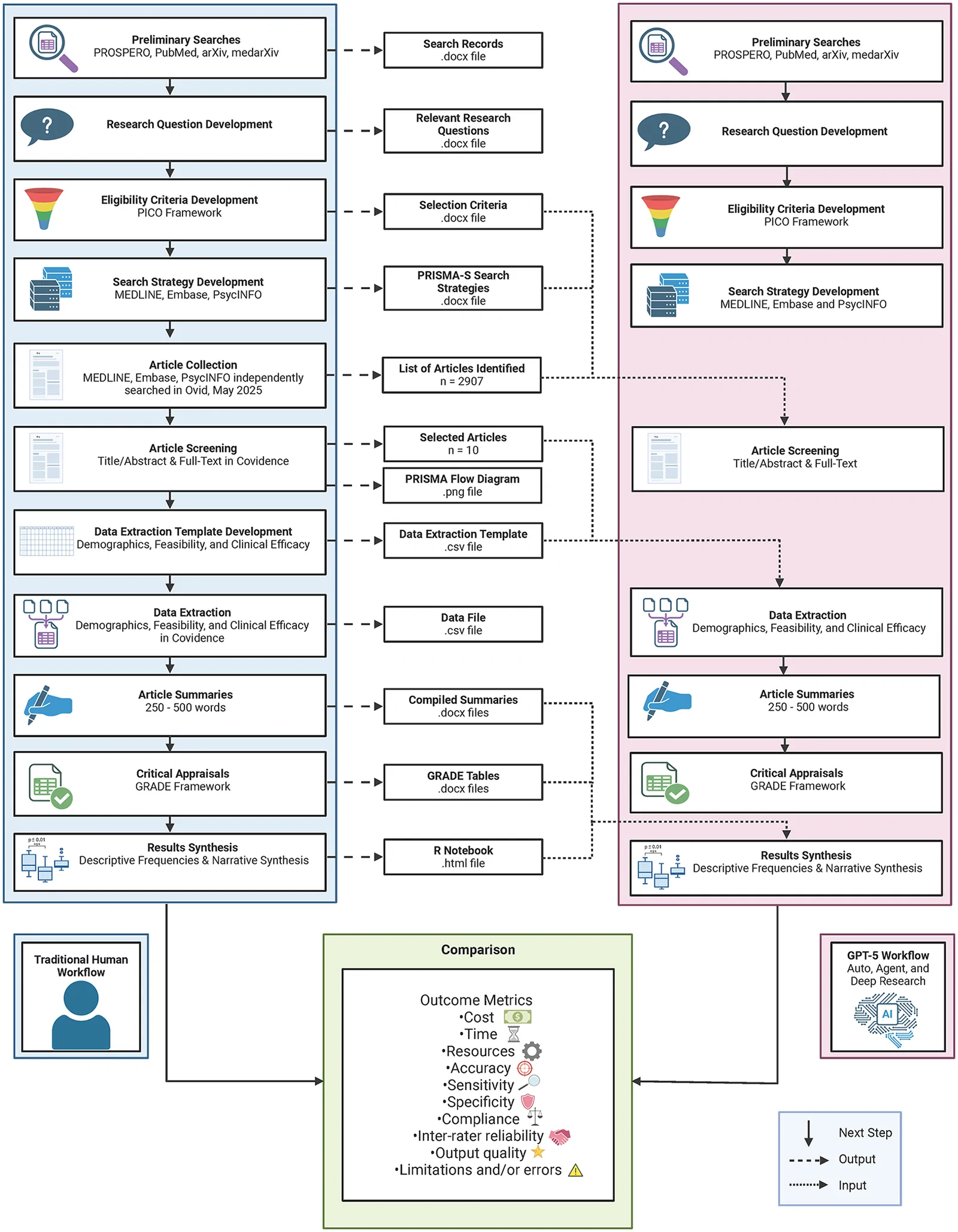
Stage-matched SLR workflows for human researchers and GPT-5 mode variants (Auto, Agent, and Deep Research). Created in Biorender https://biorender.com/r1l6x09.

As shown in Fig 1, the GPT-5 and human workflows executed nine main tasks in line with the Preferred Reporting Items for Systematic Reviews and Meta-Analyses (PRISMA) 2020 guidelines [48]. Human researchers trained in SLR processes for psychiatry research completed each task blinded to GPT-5 mode variant outputs. The first author implemented the LLM workflows (by entering the pre-constructed prompts into the ChatGPT user interface task-by-task, S2 File).

Article search and collection were intentionally conducted as part of the human-led workflow due to current limitations in ChatGPT’s ability to comprehensively and reproducibly execute literature retrieval to systematic review standards [12,49]. At present, conversational LLM interfaces demonstrate known limitations related to subscription-based database access, paywalled full-text retrieval, reproducible query execution, transparent indexing, and comprehensive recall of eligible studies, making autonomous deployment for systematic review searching insufficiently validated. Consequently, validated Ovid searches of MEDLINE, Embase, and APA PsycINFO conducted by trained researchers were used to establish a standardized article pool for downstream GPT-5 evaluation. This design minimized propagation of known retrieval error, ensured consistent benchmarking across mode variants, and aligned with the study’s intended focus on empirically evaluating key downstream SLR tasks for which preliminary evidence supports at least partial feasibility of generative AI implementation (see S1 Text).

### Materials & procedure

#### Human-led workflow.

The human-led workflow is detailed in the review results article and summarized in S2 Text. In brief, the first author conducted preliminary scoping searches (PROSPERO, arXiv, medRxiv, PubMed) and developed the research questions and eligibility criteria. Search strategies were designed in consultation with a knowledge synthesis expert and executed in Ovid across MEDLINE, Embase, and APA PsycINFO for articles published between January 1, 2000 and May 23, 2025, with screening and data extraction performed in Covidence under blinded conditions. Two human reviewers independently summarized studies, appraised evidence quality for each study using the Grading of Recommendations, Assessment, Development and Evaluation (GRADE) framework, and produced a narrative synthesis followed by first-author consensus approval, with all human-led tasks completed prior to and blinded from GPT-5 mode variant outputs.

#### LLM-assisted workflow.

Standardized prompt generation: The LLM-assisted workflow was structured using the Role, Instruction, Steps, End Goal, and Narrowing (RISEN) prompting framework, which has been applied in other psychiatry research [50,51] and SLR workflows [52]. RISEN offers a structured approach that balances structure, flexibility, and task alignment across the multi-stage SLR process. Compared to other prompting methods (e.g., few-shot or chain-of-thought prompting), which tend to emphasize only one of these major features, RISEN integrates structured reasoning with explicit goals and scope constraints, making it approachable for healthcare researchers and clinicians, and well-suited for complex, multi-stage tasks such as SLRs. For the LLM workflow, RISEN prompts were designed to emulate human-like decision-making and align outputs with SLR standards.

Systematic prompts for SLR training and the nine tasks outlined in Fig 1 were prepared by a psychiatry researcher experienced in conducting SLRs and a team of prompt engineers (S2 File). Paralleling the human-led process, RISEN prompts guided the execution of preliminary scoping searches across PubMed, arXiv, medRxiv, and PROSPERO databases, with the LLM instructed to produce a list of relevant published, pre-print, and registered reviews on the domain topic. Thereafter, LLMs were prompted to generate novel research questions for innovative SLRs, as well as PICO-aligned eligibility criteria and PRISMA-S structured search strategies for three article databases. Next, LLM prompts guided the conduct of two-stage article screening (title/abstract followed by full-text) based on the list of relevant articles identified by human-led searches in Ovid and human-derived eligibility criteria. Data extraction for the target articles (based on human consensus; N = 10) was then facilitated by prompts guiding the LLM to extract study metadata, methodological design features, sample descriptors, intervention characteristics, and outcomes into a structured format based on the uploaded (human-derived) data extraction template. RISEN prompts then instructed brief narrative summary generation (250–500 words) for each target article, capturing key results and methodological features relevant to the review topic. Prompts also facilitated the critical appraisal of evidence quality and risk of bias for each target article using the GRADE framework. Finally, the LLM was prompted to synthesize results (descriptive statistics for study characteristics and narrative synthesis of findings) across target articles.

Prompt implementation: To reduce procedural variability, RISEN prompts were pre-constructed prior to workflow implementation and applied consistently across mode variants, with deviations documented and limited to a maximum of two prompt attempts per task. The first author implemented the LLM-assisted workflow (Fig 1) in the ChatGPT user interface in October 2025 (OpenAI, 2025 release) using a ChatGPT Plus account. All LLM workflows were executed after the human-led process was complete, thus allowing for comparison to a frozen output dataset (rather than an evolving process). No back-end settings (e.g., temperature) were modified.

The workflow was implemented using three GPT-5 mode variants: 1) *Auto mode*: the default GPT-5 model selected for optimized speed, quality, or cost for the task and automated thinking time; 2) *Agent mode*: Auto mode with the Agent feature selected; and 3) *Deep Research mode*: Auto mode with the Deep Research feature selected. Mode variants were carried out sequentially: first Auto mode, second Agent mode, and third Deep Research mode. A new conversation environment (chat) was created for each mode variant, and RISEN prompts for the nine SLR tasks (S2 File) were entered into the ChatGPT user interface task-by-task (Fig 1). Thus, memory for each mode variant’s workflow was maintained internally (i.e., meaning was retained within each mode variant workflow), but not externally (between mode variants), thereby ensuring outputs were not influenced by memory of retrospective workflows.

In cases where additional prompting was required to execute the task, the researcher provided the necessary details and documented these cases in their research notes. If a task was not executed as directed on the first prompt attempt, the researcher provided feedback to the model and re-prompted for the same SLR task. If an SLR task could not be carried out after two attempts, it was noted as incomplete.

### Analytic strategy

#### Narrative summaries.

Human researchers blind to the human-led workflow process reviewed the GPT-5 mode variant outputs for each SLR task, noting key attributes of content, compliance, scientific rigour, and reliability. Observational notes were compiled and synthesized to identify similarities, differences, errors, and hallucinations in the outputs. Descriptive narrative summaries of overarching patterns across tasks and mode variants are reported.

#### Compliance.

Compliance of GPT-5 mode variant outputs was evaluated using a two-component framework assessing 1) adherence to prompt instructions and workflow execution requirements; and 2) alignment with PRISMA systematic review reporting standards. This combined approach was intended to evaluate not only whether GPT-5 mode variants successfully executed the requested SLR tasks, but also the extent to which outputs aligned with established methodological reporting expectations for SLRs.

GPT-5 mode compliance with RISEN prompt instructions was qualitatively classified for each SLR task based on how well it followed prompt instructions, task steps, and narrowing conditions (detailed in S2 File). Two researchers blinded to the human-led SLR workflow independently reviewed the GPT-5 outputs and categorized prompt compliance as *high, good, moderate, satisfactory,* or *poor*. High compliance indicated strong alignment with prompt instructions, steps, and narrowing conditions. Good compliance indicated strong task completion but included minor deviations (e.g., incorporating external sources, elaborating beyond instructions, partially completing adjacent sub-tasks). Moderate compliance indicated fair adherence, although the model occasionally deviated from intended commands, required additional prompting, or incorporated unrelated content. Outputs were categorized as satisfactory when some adherence to prompt instructions, steps, and/or narrowing was observed but errors, omissions, or incomplete task execution were present. Poor compliance indicated weak-to-no alignment with prompt instructions, substantial deviation from intended commands, inability to complete the task, or the need for substantial prompt refinement. The first author reviewed all ratings and approved consensus classifications.

To evaluate reporting completeness, transparency, and methodological alignment with SLR standards, all GPT-5 task outputs were assessed against relevant items from the (PRISMA) 2020 statement [48]. Checklist items were mapped a priori to relevant workflow stages according to their intended methodological purpose. For example, eligibility criteria development was mapped to PRISMA Item 5 (inclusion and exclusion criteria), whereas article screening was evaluated against Items 8 (selection process), 16 (study selection results), and 17 (study characteristics). When checklist items were relevant to more than one task (e.g., Item 6, information sources, and Item 7, search strategy, are both relevant to Preliminary Searches and Search Strategy Development tasks), the item was assessed independently for each task output. Because the Search Strategy Development task instructions specified alignment with the PRISMA for Searching (PRISMA-S) standards [53], these task outputs were assessed against relevant items of the primary PRISMA checklist, as well as the PRISMA-S extension. Checklist items not relevant to the current modular workflow design (e.g., citation searching, search updates) or not reflected in prompt instructions (e.g., total records, deduplication) were excluded from evaluation. GPT-5 mode variant outputs were reviewed and evaluated for the degree to which relevant checklist items were explicitly reported in the output. Compliance was operationalized using a structured ordinal framework: 0 = not reported/absent, indicating no meaningful evidence of the checklist item; 1 = partially reported, indicating incomplete, indirect, or minimally sufficient reporting; and 2 = fully reported, indicating explicit and sufficiently complete reporting consistent with PRISMA expectations. Ratings focused on the degree to which reporting elements were directly observable in GPT-generated outputs rather than inferred from successful task execution or assumed procedural implementation.

#### Accuracy, sensitivity, and specificity.

Signal detection theory [22,39,54] was applied as a standardized comparative benchmarking framework to evaluate GPT-5 mode variant outputs relative to the consensus-based human reference workflow; however, operationalization varied according to task structure and evaluability. Binary classification tasks (e.g., title/abstract and full-text screening) permitted conventional inclusion/exclusion modelling, whereas conceptual, narrative, or structured output tasks (e.g., research question development, eligibility criteria, search strategy development) required task-specific operational definitions based on correspondence between human-identified and GPT-generated elements. Hits were defined as GPT-generated outputs corresponding to human consensus-reference elements, false alarms as GPT-generated outputs not supported by the human workflow, and misses as relevant elements omitted by GPT-5. When a negative comparison class was operationally definable, correct rejections were defined as mutual exclusion of irrelevant elements (e.g., human consensus and GPT-5 exclusion of an irrelevant article during full-text screening). Full stage-specific operational definitions and scoring rules are provided in S3 Text. Accordingly, signal-detection metrics (i.e., hits, misses, false alarms, and correct rejections) should be interpreted as indicators of relative alignment with human workflow outputs rather than equivalent measures of methodological competence across heterogeneous SLR stages.

Accuracy was indexed as the proportion of correct items (hits and correct rejections) and, when possible, d′ (Equation 1).


$$\begin{array}{c} d\prime \;=\;z(proportion\;of\;hits)-z(proportion\;of\;false\;alarms) \end{array}$$ (1)

Sensitivity (Equation 2) quantified GPT-5 mode variant ability to identify relevant cases and, when possible, specificity (Equation 3) indexed identification of irrelevant cases [32]. A threshold of >80% was used to indicate performance approximating standard human-reviewer workflows, in line with validated estimates of human error rates for systematic reviews in psychiatry [55]. Specificity was only calculated for tasks with a clearly defined group of non-relevant items (e.g., screening, data extraction, critical appraisal), whereas tasks lacking a meaningful negative class (e.g., research question development, search strategy development, narrative summarization) were treated as non-calculable.


$$\begin{array}{c} sensitivity\;=\;\frac{hits}{hits\;+\;misses} \end{array}$$ (2)

$$\begin{array}{c} specificity\;=\;\frac{correct\;rejections}{correct\;rejections\;+\;false\;alarms} \end{array}$$ (3)

#### Bounded reclassification sensitivity analyses.

Bounded reclassification sensitivity analyses were conducted for article screening tasks to evaluate the robustness of GPT-5 performance estimates compared to varying assumptions regarding uncertainty in the consensus-based human reference standard. Screening tasks were selected because they involve binary inclusion/exclusion classifications with clearly operationalized disagreement structures amenable to signal-detection modelling and because errors at this stage have disproportionate downstream implications for subsequent SLR tasks. Reclassification analysis was conducted under two possible scenarios: (1) the human benchmark was treated as an approximate reflection of published medical SLR performance standards (~80% benchmark fidelity); or (2) an absolute ground truth with no benchmark error (0% error assumption). Scenarios were modelled with 10% and 20% benchmark uncertainty by iteratively reclassifying a proportion of discordant classifications as potential human omissions (potentially correct GPT identifications the human workflow missed: false alarms ◊ hits) or misclassifications (potentially ambiguous/borderline GPT identifications the human workflow excluded; misses ◊ hits), after which sensitivity, specificity, and accuracy were recalculated.

#### Hallucination monitoring and verification.

Hallucination monitoring was conducted throughout comparative benchmarking using pre-specified operational definitions tailored to each SLR task. Hallucinations were defined as fabricated or unsupported outputs not verifiable within source manuscripts, structured prompts, human consensus-reference materials, or provided article sets. Examples include invented study characteristics, unsupported findings, inaccurate attribution of study content, fabricated quantitative values, and unsupported narrative claims. Across tasks, observed output inaccuracies were categorized into a structured error taxonomy comprising: (1) factual fabrication/hallucination (unsupported or invented content); (2) omission errors (failure to identify relevant content); (3) misclassification (incorrect inclusion/exclusion or rating decisions); (4) misinterpretation (incorrect representation of source content); (5) over-inclusion (inclusion of unsupported or irrelevant information); and (6) higher-order reasoning inaccuracies (logical or interpretive errors despite accurate source information). GPT-generated outputs underwent single-pass review by trained researchers during comparative evaluation, followed by second-pass verification by the first author against source titles, extracted data files, and consensus-reference materials. Where uncertainty arose regarding hallucination classification, final determination was made by the first author.

#### Inter-rater reliability.

Cohen’s kappa (κ) [56] was used to index inter-rater agreement between human consensus and the GPT-5 mode variants (Equation 4). In this way, observed agreement between the human consensus-reference standard and GPT-5 workflows (*P*_*r*_) was evaluated relative to expected chance agreement (*P*_e_) to estimate the relative consistency of decision-making across human researchers and GPT-5 mode variants.


$$\begin{array}{c} \kappa \;=\;\frac{Pr(a)\;-\;Pr(e)}{\text{1}\;-\;Pr(e)} \end{array}$$ (4)

#### Article summary metrics.

RStudio (version 2025.09.2-418) was used to conduct statistical analyses with a significance threshold of *p <* .05.

Word counts for each article summary were tabulated and analyzed using a one-way analysis of variance (ANOVA) with Article Generator (human researcher, Auto, Agent, Deep Research) as the sole between-group factor. To further evaluate article summarization performance, four researchers with experience conducting systematic reviews blindly assessed article summaries generated by human researchers and GPT-5 mode variants. Prior to evaluation, reviewers were provided with the ten target studies included in the SLR and instructed to read each article in depth to familiarize themselves with primary objectives, methodological approaches, findings, and scientific conditions relevant to VR exergaming in depression. This calibration procedure was implemented to ensure reviewers possessed sufficient contextual familiarity to evaluate summary accuracy and plausibility while minimizing superficial or impressionistic judgements. Following calibration, reviewers completed a structured assessment consisting of 47 pseudo-randomized article summaries presented in blinded order. For each summary, reviewers were asked to: 1) rate overall summary quality and accuracy using a Likert scale from 0 (incorrect) to 10 (excellent), 2) identify the perceived generator source (human researcher, ChatGPT, or unsure), and 3) indicate confidence in their generator attribution decision using a Likert scale from 0 (no confidence) to 10 (absolute confidence).

Descriptive statistics (means, standard deviations) for summary quality ratings were computed across raters for each generator category and analyzed using a one-way ANOVA with Article Generator (4 levels) as the independent variable. When omnibus effects reached statistical significance, post hoc pairwise comparisons were conducted to identify between-group differences. For source attribution analyses, reviewer judgements were coded relative to the known generator (correct vs. incorrect attribution), and descriptive proportions were calculated across generator categories to characterize patterns of human–AI distinguishability. Inter-rater reliability for summary quality evaluations was assessed using average-measures and consistency intraclass correlation coefficients (ICC) derived from two-way models to estimate agreement and consistency among expert reviewers.

## Results

### Time and cost efficiency

The time and personnel resources required to complete each SLR task for human researchers and GPT-5 mode variants are detailed in S1 Table. Overall, the nine-task human-led SLR workflow required approximately 176 hours over six months (May–October 2025). Early-stage tasks were completed most rapidly (5–13 hours), whereas search strategy development required up to 22 hours, and article screening constituted the primary bottleneck (approximately 55 hours across six reviewers). Later multi-rater tasks, including data extraction, article summarization, and critical appraisal, required moderate time investment (18–25 hours), with narrative synthesis completed within 11 hours. Personnel costs were minimized by allocating time-intensive tasks to trained student researchers, allowing senior investigators to focus on consensus decisions, synthesis, and oversight, resulting in an estimated total cost of $1093.29 CAD.

In contrast, all GPT-5 mode variant outputs were generated within three days using a single ChatGPT Pro subscription, with a cumulative cost of $167.75 CAD (including personnel expenses). Processing time increased with mode complexity: Auto mode completed all tasks in approximately 7 minutes, Agent mode in 30 minutes, and Deep Research mode in 3.6 hours. However, observed increases in processing time across progressively more computationally intensive GPT-5 modes did not consistently correspond with proportional improvements in task performance. Deep Research most closely mirrored the human workflow’s time distribution, with article screening (33 minutes), data extraction (77 minutes), and article summarization (44 minutes) being the most time-intensive stages. Notably, these latter tasks also corresponded to weaker or incomplete outputs, particularly for data extraction and article summarization.

### Qualitative observations

A general summary and key takeaways of GPT-5 mode variants’ performance across the nine SLR tasks, benchmarked to the human-led workflow, are presented in Table 1 and elaborated below.

**Table 1:** Stage-matched comparison of GPT-5 mode variant performance across SLR tasks relative to the human-led workflow.

SLR Stage	Human Benchmark	GPT-5 Auto	GPT-5 Agent	GPT-5 Deep Research	Key Takeaway
**Preliminary Searches**	Comprehensive, targeted	Limited retrieval; conservative	Over-inclusive; retrieved irrelevant items	Structured but incomplete	GPT-5 implementation is feasible but less precise than humans
**Research Question Development**	Clinically grounded, scoped	Aligned but high-level	Broader, exploratory	Most structured and rationale-driven	GPT-5 is strong for ideation and gap identification
**Eligibility Criteria**	Fully operationalized	Clear but broad	Moderately detailed	Most operationalized	Deep Research best approximates human rigour
**Search Strategy Development**	Librarian-validated, exhaustive	Structurally correct, limited MeSH depth	Broad synonym expansion	PRISMA-S aligned but incomplete	GPT-generated strategies need expert refinement
**Title/Abstract Screening**	Balanced sensitivity and specificity with consensus	Over-exclusion (low sensitivity)	Over-inclusion (low specificity)	Most balanced, approximating human performance	GPT error profiles vary by mode
**Full-Text Screening**	Balanced sensitivity and specificity with consensus	Poor sensitivity	Moderate sensitivity	High sensitivity	Deep Research closest to human decisions
**Data Extraction**	Structured, complete, and consensus-based	Moderate accuracy for categorical fields and identifying missing data; poor performance on quantitative outcome extraction	Improved from Auto, but remains biased	Largely incomplete	Data extraction remains a major limitation of all modes
**Article Summaries**	High-quality, detailed, consistent structure, and within word limits	Concise, less detailed, and significantly below word count	Mixed descriptive/interpretive, and significantly below word count	Most synthesis-ready and within word limits, but incomplete outputs	GPT summaries approach human quality but lack detail
**Critical Appraisal (GRADE)**	Full GRADE assessments with sensitivity to cases of low evidence	Partial GRADE, biased severity	Table-based, biased severity	Could not execute task	GPT-generated GRADE appraisals biased towards high evidence and severe downgrading
**Results Synthesis**	Narrative + quantitative (study design and demographic descriptives)	Partial narrative synthesis + quantitative descriptives (values only) with a tendency to report median values	Partial narrative synthesis + more structured quantitative descriptives with observations and interpretation	Comprehensive human-like narrative synthesis + manuscript-ready paragraphs and tables reporting quantitative descriptives	GPT is consistently useful for narrative synthesis, with quantitative descriptive analysis dependent on mode variant

#### Task 1: preliminary searches.

All GPT-5 mode variants completed constrained preliminary scoping searches without hallucinations and, consistent with human reviewers, identified no relevant preprints on arXiv or medRxiv. In PROSPERO, only GPT-5 Agent identified the current review registration, along with several non-relevant records; Auto and Deep Research identified none. In PubMed, human reviewers identified eight relevant systematic reviews, of which all GPT-5 modes correctly identified two. Agent mode retrieved additional (but largely irrelevant) reviews, whereas Auto and Deep Research retrieved fewer records with clearer exclusion rationales. Deep Research produced the most structured, eligibility-aligned summaries, while Agent outputs required greater post-hoc filtering.

#### Task 2: research question development.

Given the limited literature in the domain, the human researcher framed research questions strictly around early-stage evaluation, focusing on implementation feasibility and treatment potential, with secondary questions addressing physical activity type, motivation and usability, comparative performance, severity, and dosage response. All GPT-5 mode variants successfully generated novel, evidence-based research questions aligned with these priorities. GPT-5 Auto used a stepwise approach to identify key gaps and propose five conceptually distinct questions, whereas Agent and Deep Research applied streamlined gap-identification approaches, generating seven and four primary questions, respectively. Across modes, LLM-generated questions captured feasibility and preliminary efficacy while also extending into mechanistic, comparative, and multidisciplinary domains. Deep Research provided the most structured rationales and exemplars.

#### Task 3: eligibility criteria development.

Consistent with the PRISMA human-led process, all GPT-5 mode variants generated inclusion and exclusion criteria appropriate for an SLR using the PICO framework. All modes correctly specified depression populations, immersive VR interventions, mixed feasibility and symptom outcomes, broad comparator types, and key exclusions (e.g., non-immersive exergaming, VR without exercise, healthy-only samples), although age criteria were broader than the research team’s adult-only focus. GPT-5 Auto most closely aligned with the team’s publication year restriction (≥2000) and produced clear but broad criteria. Agent mode provided no date guidance but did offer greater detail without full structure. Alternatively, Deep Research mode was overly restrictive (≥2010) but generated the most operationalized criteria and uniquely prompted clarification on population severity, immersion requirements, and inclusion of feasibility-only studies.

#### Task 4: search strategydevelopment.

All GPT-5 mode variants generated PRISMA-S compliant search strategies by mapping MeSH and keyword terms with Boolean operators. All modes captured higher-order concepts and truncation conventions (e.g., depress*), but frequently omitted nuanced or nested terms identified by human experts, with Agent mode producing the most comprehensive strategy through broader synonym expansion. Search implementation differed by mode: Auto and Agent produced the most immediately usable strings (including English-language limits), Agent uniquely specified duplicate removal, and publication-year restrictions were applied in Auto and Deep Research modes.

#### Task 5: article screening.

GPT-5 Deep Research produced the most human-like approximation of article screening, executing a balanced title/abstract screening process with justified eligibility decisions and a transparent, defensible screening record. Although Deep Research was more conservative on title/abstract screening than the research team (only identifying 25% of articles passed by the research team), it was the only GPT-5 mode able to correctly identify 90% of target articles, only producing one false alarm. In contrast, GPT-5 Auto adhered too strictly to the eligibility criteria at title/abstract screening without accommodating borderline or mixed-design studies, resulting in over-exclusion of all articles. Agent mode demonstrated the opposite pattern, allowing studies with partial relevance, adjacent constructs (e.g., serious games, rehabilitation VR), or weak alignment to depression outcomes to continue into full-text review, thereby shifting the sensitivity-specificity balance toward inclusivity relative to the human workflow. Agent mode was also the only variant that failed to produce a PRISMA flow diagram.

#### Task 6: data extraction.

Alignment with the human data structure varied across GPT-5 mode variants, with persistently high error rates. Deep Research showed the poorest coverage, extracting only 11% of predefined outcomes. In contrast, Auto and Agent approximated human performance for low-level categorical variables explicitly reported in the full text (e.g., comparators, total sample size, age) and correctly rejected absent data, but diverged on higher-order contextual variables, nuanced methodological details, and quantitative metrics, often defaulting to vague summaries or generic inferences. Agent mode demonstrated modest gains over GPT-5 Auto for blinding and depression severity identification, but introduced systematic biases (e.g., duplicated sample sizes, inferred comorbidities). Furthermore, both modes required additional prompting to complete extraction and appropriately handle formatting and missing data.

#### Task 7: article summaries.

Akin to human researchers, Auto and Agent modes generated article summaries for all target articles; however, Deep Research mode only generated summaries for eight articles. Variation between mode variants was observed for descriptive reporting and interpretive commentary balance: Auto mode generated concise, high-level quality summaries, Agent mode blended descriptive reporting with interpretive commentary, and Deep Research mode provided the most synthesis-ready outputs by incorporating quantitative detail and outcome classification.

#### Task 8: critical appraisals.

Deep Research mode did not complete the critical appraisal task, despite multiple attempts and clarification steps, indicating a breakdown in task adherence. GPT-5 Auto provided GRADE component ratings for each study in bullet-point format and a summary of findings table, whereas GPT-5 Agent most closely approximated human process and exported a document with ratings compiled in the table template (S3 File). GPT-5 Auto and Agent appraisals of seriousness aligned fairly well with the researchers’ consensus, although the LLM tended to provide more serious ratings (downgrades) than humans. Both mode variants were also sensitive to high-quality evidence ratings (correctly capturing 86% of instances identified by human researchers); however, performance was more distributed for very low-to-moderate ratings.

#### Task 9: results synthesis.

GPT-5 Auto was able to synthesize study frequencies and age descriptives comparably to human researchers; however, a significant proportion of errors (S2 Table) were attributed to qualitatively listing categories without counts or providing median values where human researchers had specified means. In contrast, most descriptive outcomes generated by GPT-5 Agent and Deep Research modes were identical to those produced by human researcher consensus, with minor discrepancies between modes consistent with Agent mode’s prioritization of sensitivity relative to Deep Research mode’s elevated specificity.

### Hallucination monitoring and verification

During structured hallucination auditing, no observable instances of fabricated study-level content were identified across evaluated tasks under the constrained conditions of the workflow. Observed inaccuracies instead appeared more commonly related to omission, over-inclusion, misclassification, or higher-order reasoning errors.

### Compliance

**Fig 2:**
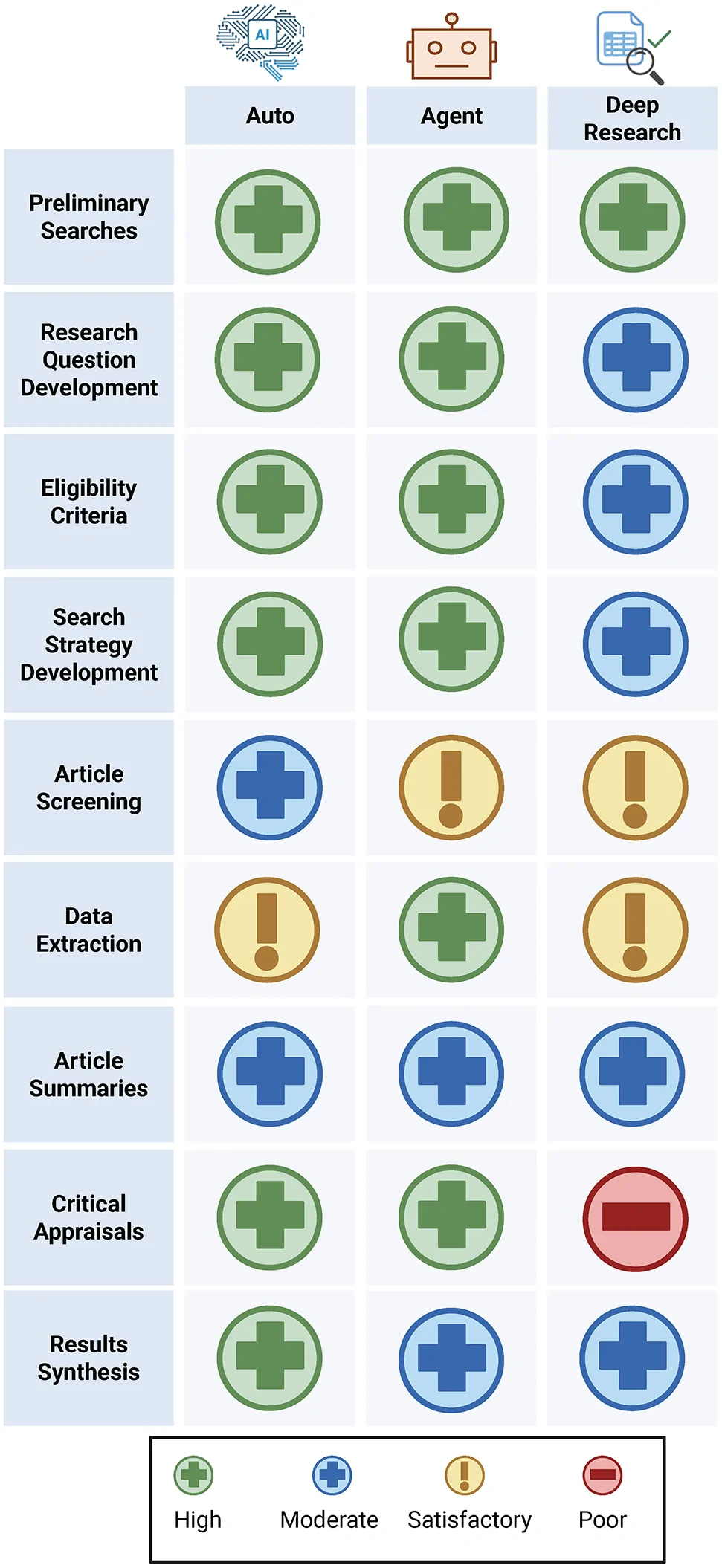
Qualitative reliability of the three GPT-5 mode variants (Auto, Agent, and Deep Research) outputs across the SLR workflow. Created in BioRender. https://BioRender.com/pcwuxzq.

As shown in Fig 2, the GPT-5 Auto and Agent models were the most compliant across tasks, with high compliance observed for seven of nine SLR tasks. The Deep Research model, however, often required additional prompting and human input to execute commands, yielding moderate or satisfactory compliance for all tasks except Preliminary Searches. It should also be noted that Deep Research mode erroneously reproduced Research Question Development outputs for two tasks (Article Screening and Critical Appraisals) and was unable to complete the Critical Appraisal task, despite a second prompt attempt.

Detailed compliance with PRISMA [48] and PRISMA-S [53] reporting standards is summarized in Tables 2,3.

**Table 2:** Evaluation of GPT-5 mode variants’ output compliance with PRISMA (2020) SLR standards.

SLR Task	Purpose	Relevant PRISMA 2020 Checklist Items	GPT-5 Compliance		
			*Auto*	*Agent*	*Deep Research*
Preliminary Searches	Determine novelty, feasibility, and existing evidence	Item 6 (Information Sources)	1	2	1
		Item 7 (Search Strategy)	2	2	0
Research Question Development	Define review scope and objectives	Item 3 (Rationale)	2	2	2
		Item 4 (Objectives)	2	2	2
Eligibility Criteria Development	Define inclusion/exclusion criteria	Item 5 (Eligibility Criteria)	1	1	1
Search Strategy Development	Construct a reproducible search methodology	Item 6 (Information Sources)	1	1	1
		Item 7 (Search Strategy)	2	2	1
Article Screening	Study selection and exclusion	Item 8 (Selection Process)	2	0	2
		Item 16a (Study Selection Results)	2	1	2
		Item 16b (Study Selection Results)	0	0	1
		Item 17 (Characteristics of Included Studies)	1	0	2
Data Extraction	Extract study characteristics and outcomes	Item 9 (Data Collection Process)	1	1	1
		Item 10a (Outcomes)	1	2	1
		Item 10b (Other Variables)	1	2	1
		Item 17 (Study Characteristics)	2	1	2
		Item 19 (Results of Individual studies)	1	1	1
Article Summarization	Summarize the included study findings	Item 17 (Study Characteristics)	2	2	1
		Item 19 (Results of individual studies)	2	1	1
		Item 20a (Synthesis results)	1	1	1
		Item 20b (Synthesis results)	0	2	1
		Item 20c (Synthesis results)	0	1	0
GRADE Appraisal	Assess certainty/quality of evidence	Item 11 (Study Risk of Bias Assessment)	1	1	0
		Item 15 (Certainty Assessment)	1	1	0
		Item 18 (Risk of bias in studies)	2	1	0
		Item 20a (Synthesis results)	2	2	0
		Item 20c (Synthesis results)	1	1	0
		Item 22 (Certainty of evidence)	2	2	0
Results Synthesis	Integrate evidence into conclusions	Item 13a–13c & 13f (Synthesis Methods)	0	0	0
		Item 13d (Synthesis Methods)	1	0	1
		Item 13e (Synthesis Methods)	0	1	0
		Item 20a (Results of Syntheses)	1	2	1
		Item 20b (Results of Syntheses)	1	2	2
		Item 20c (Results of Syntheses)	0	1	1
		Item 23a (Discussion)	0	1	1
		Item 23b (Discussion)	0	2	1
		Item 23c-23d (Discussion)	0	0	0

**Table 3:** Evaluation of GPT-5 mode variants’ Search Strategy Development output compliance with PRISMA-S extended standards for reporting systematic review search strategies.

Relevant PRISMA-S Extended Checklist Items	GPT-5 Compliance		
	*Auto*	*Agent*	*Deep Research*
Item 1 (Database Name)	2	2	2
Item 7 (Other Methods)	2	1	2
Item 8 (Full Search Strategies)	2	2	1
Item 9 (Limits and Restrictions)	1	1	2
Item 10 (Search Filters)	0	0	0
Item 13 (Dates of Searches)	1	0	0

Overall, GPT-5 mode variant outputs demonstrate partial-to-substantial agreement with SLR reporting standards, with meaningful differences observed in reporting completeness and transparency across workflow stages. Across upstream review planning tasks, all GPT-5 modes consistently reported review rationale and objectives and partially aligned with eligibility criteria reporting standards through structured PICO-based inclusion and exclusion criteria, although operational details regarding study grouping for synthesis were generally absent (Table 2).

Notably, search strategy reporting demonstrated the greatest variability between mode variants (Tables 2,3). For this task, Auto and Agent modes produced implementation-ready database-specific search strategies incorporating Boolean operators, keyword structures, and acknowledgement of controlled vocabulary systems (e.g., MeSH, Emtree). Furthermore, Auto mode uniquely included an explicit PRISMA-S oriented documentation section specifying checklist-relevant reporting elements (e.g., information sources, recommendations for deduplication) and placeholders for researcher-confirmed procedural details (e.g., date of search execution). Alternatively, Deep Research generated more conceptually structured, but less immediately executable, search procedures. Although Deep Research demonstrated comparatively weaker alignment with implementation-ready formatting (Item 7), it provided greater methodological justification regarding search limits, topic-specific terminology (e.g., drawing on comparable published studies or reviews), and adaptation of restrictions across databases, reflected in stronger PRISMA-S alignment for reporting limits and restrictions (Item 9).

Differences were also observed for article screening-related reporting (Items 8, 16, and 17; Table 2), corresponding to the assessment of study selection procedures, screening outcomes, and reporting of included study characteristics. Auto and Deep Research modes more clearly documented screening procedures and study flow, with both generating PRISMA flow diagrams and demonstrating stronger alignment with selection process reporting standards. In contrast, Agent mode demonstrated only partial compliance for study selection reporting despite documenting record traceability. Deep Research uniquely cited excluded full-text studies, with reasons for exclusion, in the main output and demonstrated comparatively stronger reporting of included study characteristics.

Across downstream synthesis tasks, compliance with reporting standards was generally limited and appeared influenced by RISEN prompt constraints. For instance, Agent mode demonstrated comparatively stronger reporting of interpretive findings by providing dedicated conclusions and limitation sections, whereas Auto mode remained primarily descriptive, and Deep Research embedded interpretation within narrative synthesis. Collectively, GPT-5 mode variants demonstrated distinct reporting profiles relative to PRISMA standards, with Auto mode emphasizing checklist-oriented and implementation-ready reporting, Agent mode demonstrating stronger interpretive reporting, and Deep Research mode providing greater methodological explanation but comparatively less implementation-ready formatting.

### Accuracy, sensitivity, and specificity

Signal detection outcomes (hit, false alarm, miss, and correct rejection counts) are detailed in S2 Table. Because evaluated SLR tasks differed substantially in output structure and evaluability, signal-detection metrics should be interpreted within task context rather than compared directly across tasks. Accordingly, metrics are intended to index relative alignment with the consensus-based human workflow rather than absolute methodological competence. Overall, GPT-5 mode variant accuracy, sensitivity, and specificity were low-to-moderate (0.00 - 0.71) for most tasks when benchmarked to a human-consensus reference standard (Table 4).

**Table 4:** Accuracy, sensitivity, and specificity outcomes for GPT-5 mode variants (Auto, Agent, and Deep Research) relative to human researcher outputs.

Task	Accuracy			Sensitivity			Specificity		
	*Auto*	*Agent*	*Deep Research*	*Auto*	*Agent*	*Deep Research*	*Auto*	*Agent*	*Deep Research*
Preliminary Searches	0.03	0.26	0.03	0.03	0.10	0.03	–	**1.00**	–
Research Question Development	0.33	0.46	0.56	0.43	0.71	**0.83**	–	–	–
Eligibility Criteria	0.67	0.67	0.67	0.56	0.50	0.50	**1.00**	**1.00**	**1.00**
Search Strategy	0.26	0.20	0.20	0.29	0.21	0.21	–	–	–
Article Screening - Title/Abstract	**0.98**	**0.88**	**0.99**	0.00	**0.86**	0.25	**1.00**	**0.88**	**1.00**
Article Screening – Full Text	0.00	**0.97**	**0.87**	0.00	0.00	**0.90**	–	**1.00**	**0.80**
Data Extraction	0.51	0.70	0.05	0.23	0.57	0.05	**0.85**	**0.90**	0.60
Article Summaries	**1.00**	**1.00**	**0.80**	**1.00**	**1.00**	**0.80**	–	–	–
Critical Appraisals	0.69	0.58	–	0.71	0.47	–	0.67	0.69	–
Results Synthesis	0.34	0.79	0.71	0.34	**0.87**	0.72	0.33	0.50	0.64

– Specificity was not calculable due to the absence of an operationalizable negative comparison class.

When benchmarked against the human reference workflow, GPT-5 mode variants demonstrated their strongest performance in article screening and article summarisation tasks. Title/abstract screening accuracy was high (≥0.88) across models; however, this was driven largely by correct rejections, with sensitivity remaining low for Auto (0.00) and Deep Research (0.25). In contrast, Agent mode achieved higher sensitivity at this stage (0.86) with some loss of specificity (0.88). At full-text screening, Deep Research showed the most human-aligned performance, achieving high accuracy (0.87) and sensitivity (0.90), whereas Auto excluded all studies (sensitivity 0.00), reflecting an extreme specificity-focused profile.

Across upstream conceptual tasks, including research question development, eligibility criteria, and search strategy formulation, model performance ranged from low to moderate accuracy (0.20–0.67), reflecting partial conceptual alignment with human outputs but limited consistency in identifying relevant elements (sensitivity 0.21–0.71). For downstream synthesis tasks, descriptive results synthesis approached human-performance thresholds for Agent (accuracy 0.79; sensitivity 0.87) and Deep Research (accuracy 0.71; sensitivity 0.72), while data extraction and critical appraisal showed only moderate agreement with human reviewers (accuracy 0.51–0.70 and 0.58–0.69, respectively), despite relatively preserved specificity for data extraction. Collectively, these findings indicate that GPT-5 models perform best on structured screening and summarisation tasks, with diminishing reliability as methodological complexity and interpretive demands increase.

Bounded reclassification sensitivity analyses demonstrated that the relative performance patterns across GPT-5 mode variants remained broadly stable under varying assumptions of human benchmark uncertainty (S2 Table). Agent mode title/abstract screening performance was the most sensitive to assumptions regarding human benchmarking uncertainty (sensitivity: 0.63 – 0.95; specificity: 0.86 – 0.90) across simulated error scenarios. Deep Research full-text screening performance also showed modest improvements under increasing benchmark uncertainty assumptions, with sensitivity increasing by 0.04 and specificity increasing by 0.06. Alternatively, Auto mode performance was largely unchanged across scenarios due to persistently poor screening sensitivity.

### Inter-rater agreement

Substantial variability in inter-rater agreement was observed between GPT-5 mode variants and the human consensus-reference standard across SLR tasks (Table 5). Agreement was strongest for the article screening tasks, with near-perfect agreement (κ > 0.97) for Auto and Deep Research modes, and substantial agreement for Agent mode. The exception, however, was Auto mode full-text screening, which could not be executed due to the elimination of all articles at the title/abstract screening. Moderate-to-substantial agreement was observed for Eligibility Criteria Development across mode variants, with Deep Research mode yielding the strongest agreement. In contrast, Search Strategy Development demonstrated poor agreement across evaluable modes, suggesting greater divergence from human-generated search strategies. Critical appraisal tasks demonstrated moderate-to-substantial agreement for both domain-level GRADE ratings (e.g., uncertainty, precision, risk of bias) and overall evidence quality ratings, although Deep Research mode could not be evaluated due to insufficient comparable outputs.

**Table 5:** Inter-rater agreement (as measured by Cohen’s kappa, κ) in coding decisions between a human consensus-reference standard and GPT-5 mode variants (Auto, Agent, and Deep Research).

Task	Cohen’s kappa (κ)		
	*Auto*	*Agent*	*Deep Research*
Eligibility Criteria Development	0.46	0.73	0.78
Search Strategy Development	0.18	0.26	–
Title & Abstract Screening	**0.98**	**0.88**	**0.98**
Full-text Screening	–	**0.97**	**0.86**
Critical Appraisals (Domain Ratings)	0.70	0.73	N/A
Critical Appraisals (Overall Certainty)	0.65	0.70	N/A

- Specificity was not calculable due to the absence of an operationalizable negative comparison class.

N/A GPT-5 Deep Research did not produce critical appraisal outputs that could be evaluated.

* *Note:* All GPT-5o mode variants were compared against human consensus-reference decisions.

### Article summary metrics

#### Word limits.

Summaries generated by human researchers (range: 260–415, *M* = 306.25, *SD* = 59.56) and GPT-5 Deep Research (range: 272–333, *M* = 313.00, *SD* = 19.70) were highly compliant with the specified word limit. However, summaries generated by Auto (range: 73–121, *M* = 90.90, *SD* = 14.57) and Agent (range: 98–180, *M* = 136.70, *SD* = 21.61) mode variants were considerably shorter.

#### Content quality.

Expert reviewers rated Deep Research (*M* = 8.06, *SD* = 1.19) and human (*M* = 7.97, *SD* = 1.15) summaries as the highest quality, followed by Agent (*M* = 7.58, *SD* = 1.26) and Auto (*M* = 6.95, *SD* = 1.18) summaries. Significant differences were found between outputs (*F*(3,184) = 7.80, *p* < .001), with human and Deep Research (*p*s ≤ .001) summaries being rated significantly higher than Auto mode summaries. No other ratings were different from each other (*p*s ≥ .12). Notably, inter-rater reliability for summary quality ratings was low across expert evaluators using both average-measures absolute agreement (ICC[A,4] = 0.06, 95% CI −0.47 to 0.43, *p* = .38) and consistency models (ICC[C,4] = 0.06, 95% CI −0.47 to 0.43, p = .39), suggesting limited convergence in expert evaluations of narrative summary quality.

#### Source generation.

Expert reviewers were able to correctly identify human- and GPT-generated article summaries more than half of the time (58% and 61%, respectively). When looking across GPT-5 mode variants, Auto and Agent-generated summaries were both incorrectly attributed to human researchers in 48% of cases. Only Deep Research summaries were correctly identified across the majority of summaries (81% attributed to ChatGPT, 19% attributed to human researchers).

## Discussion

The present stage-matched proof-of-concept demonstrates that prompt-driven GPT-5 workflows are feasible and semi-efficacious for supporting multiple tasks required for SLRs in digital psychiatry, but with substantial task- and mode-dependent variability relative to consensus-based human reviewers. Across nine core SLR stages, performance was neither uniform nor interchangeable across GPT-5 mode variants, revealing distinct strengths, limitations, and failure profiles. Auto mode performed best for structured, implementation-oriented tasks requiring efficiency and rule-based decision-making (e.g., eligibility criteria development), but tended toward overly conservative exclusion during article screening and generated comparatively brief summaries. Agent mode demonstrated stronger performance for conceptual and interpretive tasks, including research question generation, critical appraisal, and descriptive synthesis, although it frequently prioritized sensitivity over specificity, resulting in broader inclusion patterns and occasional over-interpretation. Deep Research mode most closely approximated human reasoning for higher-order synthesis tasks, demonstrating the strongest full-text screening performance (sensitivity = 0.90), producing article summaries rated comparatively to human outputs, and generating the most comprehensive narrative synthesis, albeit with substantially longer processing times and occasional task-execution failures (e.g., missing article summaries, no critical appraisal execution). Notably, despite concerns regarding hallucination risk in scientific applications of LLMs, no fabricated study-level content was identified during structured auditing under the constrained conditions of the present workflow. However, persistent weaknesses in quantitative data extraction, nuanced methodological interpretation, and evidence quality appraisal underscore that current LLM performance remains uneven across review stages. Collectively, these findings present an adaptable, prompt-driven framework that healthcare researchers and clinicians can incorporate into their SLR workflows and highlight that AI-assisted evidence synthesis is presently best conceptualized as a modular, human-in-the-loop decision-support process in which task-specific deployment strategies are selectively matched to mode strengths, rather than as a fully autonomous end-to-end SLR system.

The principal contribution of the present study extends beyond demonstrating that LLM-assisted SLR workflows remain imperfect or require human oversight. Rather, this study provides one of the first clinically oriented, stage-matched evaluations of a prompt-driven GPT-5 workflow spanning the major stages of the SLR process within a single operational pipeline benchmarked against a consensus-based human reference workflow. In contrast to prior investigations focused primarily on isolated benchmarking tasks, engineering optimization, or backend automation environments, the present work specifically evaluates how healthcare researchers and clinicians can implement structured AI-assisted review procedures using a widely accessible conversational interface and reproducible natural-language prompting framework. To support immediate implementation and methodological transparency, empirically evaluated RISEN prompts benchmarked against a human consensus-reference workflow are provided in S2 File. In this way, the present study contributes practical translational guidance regarding workflow implementation, task-specific deployment considerations, and mode-dependent strengths and limitations relevant to real-world evidence synthesis settings.

The observed task-specific variability likely reflects fundamental differences in the methodological demands of SLR stages. GPT-5 mode variants demonstrated comparatively stronger performance for bounded and highly structured tasks with explicit decision rules, such as eligibility criteria development, article screening, summarization, and descriptive synthesis. In contrast, performance diminished for tasks requiring nuanced methodological interpretation, contextual reasoning, quantitative precision, and evaluative judgement, particularly during structured data extraction and GRADE-based critical appraisal. Although Auto and Agent modes approximated human performance for explicitly reported categorical variables and correctly identified absent information, higher-order constructs and quantitative outcomes frequently introduced inaccuracies through omission, duplication, contextual inference, or incomplete extraction. Similarly, Deep Research mode, despite stronger performance for higher-order synthesis and narrative reasoning, showed notable limitations in task adherence and quantitative extraction. These findings suggest that current LLMs remain more effective at organizing and synthesizing clearly bounded scientific information than reliably executing tasks requiring detailed methodological interpretation or dispersed numerical reasoning across studies.

Notably, observed inaccuracies were more commonly related to omission, over-inclusion, misclassification, and higher-order reasoning errors than factual fabrication. Although no observable fabricated study-level content was identified under the structured conditions of the present workflow, this finding should be interpreted within the context of a constrained prompting environment using predefined article sets, human-curated materials, and structured RISEN prompts. Consequently, concerns regarding hallucination in AI-assisted evidence synthesis may benefit from broader conceptualization beyond fabricated outputs alone to include completeness, methodological interpretation, and reasoning quality. At the same time, the absence of hallucinated articles or fabricated study-level content across GPT-5 outputs represents a meaningful improvement relative to earlier generations of LLMs and may further underscore the importance of structured prompting and explicit narrowing conditions in reducing unsupported outputs during scientific information processing. The observed weaknesses in quantitative extraction and appraisal tasks highlight an important interpretability challenge in generative LLM-assisted evidence synthesis. While the present study was not designed to formally evaluate underlying technical mechanisms, inaccuracies may plausibly reflect difficulties interpreting complex tables, integrating dispersed quantitative information across study manuscripts, or resolving contextual ambiguity in reporting formats. Future engineering-oriented work incorporating explainable AI (XAI) approaches and structured justification prompting [57–60] may help clarify how LLMs process scientific information and improve transparency surrounding extraction decisions, omitted information, and methodological reasoning.

The present findings additionally highlight meaningful mode-dependent trade-offs relevant to the implementation of AI-assisted evidence synthesis workflows. Rather than conceptualizing GPT-5 as a single uniform system, results suggest that mode variants exhibit distinct operational profiles characterized by differing sensitivity-specificity balances, reporting strengths, interpretive tendencies, and efficiency-quality trade-offs across review stages. Auto mode demonstrated stronger procedural fidelity and implementation-ready outputs for structured tasks, particularly where checklist alignment and rapid execution were prioritized. Agent mode demonstrated greater conceptual breadth and interpretive reasoning but often shifted toward higher sensitivity and broader inclusion, occasionally at the expense of specificity or adherence to task constraints. Deep Research mode most closely approximated human reasoning during higher-order synthesis tasks and generated the strongest full-text screening and narrative synthesis outputs but required substantially longer processing times than other GPT-5 modes and demonstrated occasional task-execution failures.

Importantly, increased computational depth did not consistently translate into superior performance. Although more computationally intensive modes required substantially longer processing durations, performance gains appeared highly task-dependent. Higher-order synthesis and interpretive tasks may benefit from greater interpretive depth, whereas lower-complexity or rule-based tasks were completed comparably, and occasionally more effectively, by lower-latency modes. Similar mode-dependent distinctions emerged in reporting behaviour and communication style. Evaluation against PRISMA [48] and PRISMA-S [53] reporting standards demonstrated that Auto mode emphasized implementation-ready and checklist-oriented reporting, Agent mode demonstrated comparatively stronger interpretive and conclusion-oriented reporting, and Deep Research provided greater methodological explanation and transparency surrounding review decisions, despite weaker implementation readiness. Likewise, expert reviewers demonstrated only modest ability to distinguish human- from GPT-generated article summaries, with Auto and Agent summaries frequently misattributed to human researchers and Deep Research outputs more readily recognized as AI-generated despite comparable quality ratings. Collectively, these findings suggest that optimal implementation of AI-assisted SLRs may ultimately require task-specific mode-dependent strategies rather than reliance on a single GPT-5 mode variant across the full workflow.

From an implementation perspective, the present study was intentionally designed as a modular decision-support framework rather than an end-to-end autonomous workflow. Healthcare researchers and clinicians conducting systematic reviews may selectively implement some, many, or all evaluated tasks according to methodological appropriateness, confidence in model performance, and local workflow needs. For example, article searching may remain librarian-supported or researcher-led, whereas prompt-driven assistance may be incorporated for screening, summarization, synthesis, or critical appraisal support. Accordingly, the present findings were intended to inform task selection and implementation decisions rather than advocate replacement of established systematic review methodology. This implementation approach also aligns with emerging frameworks for trustworthy AI deployment in healthcare and scientific contexts [37,57], which emphasizes iterative processes to govern, map, measure, and manage AI-related risks. Within AI-assisted evidence synthesis, structured human oversight may serve as a practical risk mitigation strategy by identifying omissions, misclassification, reasoning inaccuracies, and unsupported outputs before these errors propagate into systematic reviews, clinical recommendations, or downstream scientific interpretation. In this regard, human-in-the-loop workflows may represent not only a methodological preference but also a pragmatic safeguard for responsible implementation of generative AI in healthcare research settings.

**Fig 3:**
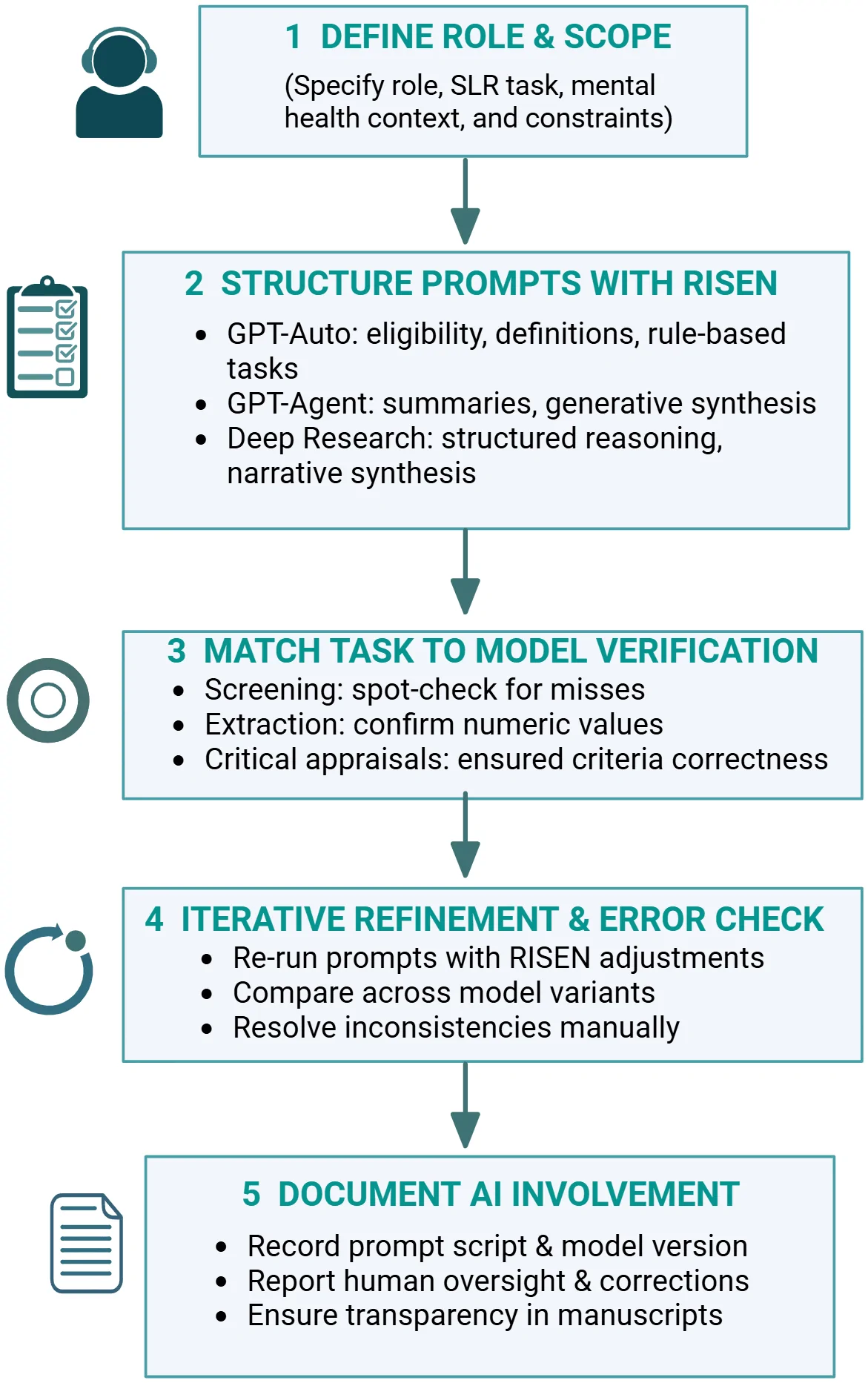
Recommendations for implementing prompt-driven generative AI workflows in ChatGPT for SLRs in mental health research. Created in BioRender. https://BioRender.com/97tsqdb.

Based on the performance patterns observed in this stage-matched comparison, implementing prompt-driven generative AI workflows for SLRs in psychiatry requires a structured, task-specific, and transparently governed approach. We propose a modular decision-making framework for healthcare researchers and clinicians integrating prompt-driven AI workflows into psychiatry research or practice (Fig 3).

Principally, we recommend that researchers begin by explicitly defining the reviewer role, SLR task, psychiatric context, and methodological constraints to anchor model behaviour and reduce ambiguity. Prompts should then be systematically structured, with deliberate alignment between task demands and mode variants. Crucially, each SLR task must be matched to explicit verification procedures, including spot-checking for missed studies during screening, confirmation of numeric values during data extraction, and correct application of appraisal frameworks such as GRADE. Iterative refinement (through prompt adjustment, cross-mode comparison, and manual reconciliation of inconsistencies) should be treated as a core component of the workflow rather than an exception. Finally, rigorous documentation of AI involvement is essential, including archiving prompts, recording model versions, and explicitly reporting human oversight and corrections to ensure transparency, reproducibility, and methodological integrity in psychiatric evidence synthesis.

To support practical implementation, the task-specific RISEN prompts used throughout the workflow are provided in S2 File as adaptable templates for future use. Rather than requiring researchers to construct prompting strategies *de novo*, these materials provide a structured starting point that can be modified according to different review questions, clinical domains, and methodological requirements. Importantly, because the RISEN prompts are platform-agnostic and based on structured natural-language instructions rather than platform-specific programming, they may facilitate methodological consistency, replication, and cross-study comparability across future AI-assisted systematic reviews in mental health and broader health sciences contexts using different generative AI platforms (e.g., ChatGPT, Copilot, DeepSeek). We anticipate that this type of standardized but adaptable prompting approach may help reduce variability in workflow implementation while lowering barriers to responsible adoption of LLM-assisted evidence synthesis methods among healthcare researchers and clinicians with limited technical expertise.

Importantly, the present findings should not be interpreted as positioning human reviewers as an infallible reference standard. Consensus-based SLR workflows remain vulnerable to omission bias, interpretive variability, reviewer fatigue, and judgement-related biases even under duplicate-review conditions [12,19]. Consistent with this perspective, bounded reclassification sensitivity analyses demonstrated that relative performance patterns across GPT-5 mode variants remained broadly stable under varying assumptions regarding human benchmark uncertainty, suggesting that observed mode-specific strengths are unlikely to be solely artifacts of imperfections in the human reference process. Consequently, disagreement between GPT-5 outputs and human consensus should not necessarily be interpreted as unequivocal model error but may instead reflect uncertainty or variability inherent to evidence synthesis itself.

The broader significance of these findings should be interpreted within the rapidly evolving digital psychiatry and precision medicine landscape. Across psychiatry and health sciences, AI-driven approaches are increasingly being integrated into digital phenotyping, passive symptom monitoring, multimodal biomarker identification, relapse prediction, clinical decision support, and personalized intervention frameworks [14,17,18]. These developments are generating increasingly large, heterogenous, and rapidly evolving evidence bases that challenge the feasibility of conventional review methodologies. Because systematic reviews underpin clinical recommendations, guideline development, and evidence-based psychiatric care, delays in evidence synthesis and appraisal may impede timely clinical translation of emerging findings [13]. In this context, prompt-driven LLM workflows may represent a potentially important translational support tool for augmenting evidence synthesis capacity among healthcare researchers and clinicians working within rapidly expanding domains. At the same time, the present findings reinforce that implementation in higher-stakes healthcare contexts should remain selective, transparent, and supported by sustained human verification, particularly for review stages requiring quantitative precision or nuanced methodological interpretation. Furthermore, although the present study only involved publicly available published literature and did not process patient-level psychiatric or identifiable health data, future implementation of LLM-assisted workflows in digital psychiatry and clinical decision-support settings will require careful consideration of privacy-preserving infrastructures, institutional governance, and secure deployment environments for sensitive health information. From a governance perspective, the present human-in-the-loop workflow may also be conceptualized as a structured risk-management strategy aligned with emerging frameworks for trustworthy AI implementation, such as the NIST AI Risk Management Framework [37]. Specifically, consensus-based verification, hallucination monitoring, task-specific benchmarking, and selective deployment of GPT-5 mode variants according to observed strengths and limitations reflect practical mechanisms for identifying, measuring, and mitigating risks associated with omission, misclassification, unsupported outputs, and downstream error propagation in AI-assisted scientific workflows. Consistent with NIST principles, these findings support a model of sustained human accountability and context-sensitive implementation rather than autonomous deployment.

The present findings are broadly consistent with, and extend, prior evaluations of AI-assisted SLRs. Previous studies have shown that machine learning and LLM-based tools can meaningfully reduce screening burden and, under constrained conditions, demonstrate substantial agreement with human reviewers [12,19,23,28,33,34,40–42,61]. Our findings corroborate these efficiencies while extending the literature in several important ways. First, unlike many prior studies focused on isolated benchmarking tasks, the present work evaluated nine major SLR stages within a single stage-matched workflow compared to the field standard: a blinded, consensus-based human process. Second, the present study examined GPT-5 performance within a widely accessible conversational interface (ChatGPT) rather than an engineering-oriented or API-dependent environment, thereby improving ecological validity for healthcare researchers and clinicians with limited computational expertise. Third, findings demonstrate that apparent performance gains may obscure qualitatively distinct failure profiles across GPT-5 mode variants, reinforcing that high agreement metrics alone should not be interpreted as evidence of reliable autonomous implementation.

Interestingly, expert reviewers demonstrated only modest ability to distinguish human- from GPT-generated article summaries, correctly identifying human and GPT outputs in 58% and 61% of cases, respectively. Auto and Agent outputs were frequently misattributed to human researchers, whereas Deep Research summaries were more consistently recognized as AI-generated despite receiving quality ratings comparable to human-authored outputs. These findings suggest that although GPT-generated summaries may, in some contexts, approximate human scientific writing, GPT-5 mode variants appear to produce meaningfully different stylistic and interpretive signatures that may influence transparency, perceived authorship, and implementation suitability across SLR tasks. Interpretation of article summary quality findings nonetheless warrants some caution. Inter-rater reliability agreement and consistency ICC estimates among expert evaluators were low, suggesting meaningful variability in how reviewers prioritized dimensions such as factual completeness, methodological nuance, readability, interpretability, or conciseness. Accordingly, summary quality findings are best interpreted here alongside complementary indicators, including blinded source attribution accuracy and word-count characteristics, rather than as a standalone determinant of model performance. It would be valuable for future studies involving larger reviewer pools and article samples to inform rater-level variance more directly using advanced methods such as mixed-effects modelling approaches capable of accounting for article-level clustering.

An important methodological consideration concerns the deliberate exclusion of AI-assisted article retrieval from the current evaluated GPT-5 workflow. Although literature searching and evidence collection represent foundational SLR stages, current evidence does yet support reliable deployment of conversational LLM interfaces for comprehensive article retrieval to SLR standards due to known limitations in database access, paywalled literature retrieval, reproducibility, retrieval completeness, and sensitivity to prompt formulation [12,19,20]. Accordingly, omission of this stage was intentional and methodologically conservative rather than a limitation of oversight in study design. GPT-generated search strategies demonstrated partial alignment with PRISMA-S expectations but remained less comprehensive than a librarian-supported search, particularly regarding controlled vocabulary depth, synonym breadth, and database-specific refinement. Moreover, the incomplete identification of relevant published research during GPT-assisted preliminary searches in the present study raises important concerns regarding implications for downstream error propagation if LLMs are used for full article searching and collection or as a fully autonomous pipeline. Because omissions at the retrieval stage constrain the evidentiary corpus available for all subsequent SLR tasks, incomplete search performance may systematically bias screening decisions, evidence synthesis, pooled estimates, and guideline-relevant conclusions, further supporting the present study’s methodologically conservative exclusion of autonomous LLM-assisted article retrieval from the evaluated workflow. Consequently, GPT-derived searches appear to function best as starting points for researcher refinement and execution, rather than comprehensive autonomous retrieval systems capable of independently seeding downstream SLR tasks. In practical settings, article retrieval may continue to be conducted through librarian-supported or researcher-led workflows while downstream tasks are selectively augmented using prompt-driven LLM support. Future research should evaluate retrieval performance under increasingly realistic constraints as scientific database integration, retrieval architectures, and reproducibility standards for generative AI systems continue to evolve.

Several limitations warrant consideration. First, performance was evaluated within a single review topic characterized by a relatively small but conceptually heterogeneous evidence base. Although this design facilitated detailed stage-matched benchmarking under clinically realistic conditions, the current study has limited generalizability across health science domains, methodological structures, and larger datasets. Future research should examine whether mode-dependent trade-offs and error profiles observed here represent stable characteristics of GPT-assisted workflows or vary according to evidence density, review complexity, and/or screening ambiguity. Longitudinal reproducibility research is also needed because rapidly evolving deployment environments, model updates, subscription tiers, retrieval systems, and account-level contextual factors may influence workflow outputs over time, thereby introducing important challenges for reproducibility and standardization in applied healthcare AI research. Second, GPT-5 mode variants were implemented sequentially by a single trained operator within a conversational interface. Although RISEN prompts were standardized in advance, applied to identical frozen task inputs, and implemented within separate chat environments to reduce contamination across workflows, sequential execution may nonetheless have introduced procedural bias through learning effects or evolving expectations regarding model behaviour. Conversely, use of a single trained operator reduced variability in prompting style and implementation that may otherwise confound between-mode comparisons. Future studies should incorporate randomized mode order, prompt immutability protocols, counterbalanced designs, and multiple independent operators to strengthen causal inference regarding mode-specific performance.

Third, the chat-based interface constrained reproducibility through limited parameter control, restricted access to execution logs, and the proprietary “black box” nature of conversational LLM interfaces. Consequently, underlying mechanisms contributing to observed failures – such as quantitative extraction inaccuracies and appraisal inconsistencies which may plausibly reflect challenges related to the LLM’s interpretation of complex tables, multi-step quantitative reasoning, extraction of dispersed numerical information across manuscripts, or contextual ambiguity in reporting formats – could not be directly evaluated. Future engineering-oriented investigations incorporating API-based architectures, persistent state tracking, and XAI-oriented methods may improve causal understanding of task failures while preserving structured human oversight. Relatedly, although some GPT-5 mode variants demonstrated stronger reasoning or synthesis capabilities than others, none showed reliable capacity for self-critique, error detection, or iterative correction without explicit human prompting. This reinforces that quality assurance and methodological verification presently remains a human responsibility rather than a safely delegable AI function.

Finally, although a blinded, consensus-based human workflow supported by bounded reclassification analysis served as the pragmatic reference standard in this study, current systematic review methodology lacks a truly error-free comparator standard. Consensus adjudication remains the accepted best-practice approach for evidence synthesis, however, residual disagreement and omission rates persist [12,19]. Manual evidence synthesis processes remain vulnerable to omission, interpretive subjectivity, anchoring effects, and reviewer fatigue even under duplicate-review circumstances [12,19,20,55]. Consequently, concordance metrics reported here should be interpreted as indicators of alignment with current best-practice evidence synthesis processes rather than absolute correctness. This distinction is particularly important because disagreement between GPT-5 outputs and human consensus may reflect either genuine model failure or uncertainty inherent to evidence synthesis itself. Future methodological research may benefit from moving beyond binary notions of “human-correct versus AI-incorrect” toward uncertainty-aware benchmarking approaches incorporating probabilistic inclusive modelling, adjudication panels, and external validation frameworks capable of explicitly accounting for variability in the human reference process.

As digital psychiatry and AI-enabled mental health research continue to expand, scalable approaches to evidence synthesis may become increasingly important for supporting timely clinical translation, evidence-based practice, and guideline development. The present study provides a clinically oriented AI proof-of-concept by offering empirically evaluated RISEN prompts and a structured, adaptable framework that healthcare researchers and clinicians can selectively integrate into their SLR workflow without requiring extensive technical expertise. Prompt-driven LLM workflows implemented through conversational interfaces may offer a feasible and practical decision-support approach for selected SLR tasks in rapidly evolving fields, such as digital psychiatry, when deployed within structured, human-supervised workflows. However, substantial task- and mode-dependent variability, coupled with important efficiency-quality trade-offs across GPT-5 variants, reinforces that selective implementation guided by methodological appropriateness, desired output fidelity, processing burden, and required human verification, rather than autonomous deployment, currently represents the most scientifically defensible application of LLM-assisted evidence synthesis in healthcare settings.

## Supporting information

S1 Text Review of existing evidence for AI in systematic review tasks. Narrative review of existing literature evaluating the use of AI, including LLMs, in SLR tasks. Includes a summary of prior benchmarking studies, task-specific applications across evidence synthesis stages, and currently available AI applications for AI-assisted review workflows within healthcare and broader research contexts.
(PDF)

S1 File Design and Evaluation of AI in Literature Reviews – Benchmarking (DEAL-B) checklist for standardized reporting. Completed DEAL-B checklist detailing study reporting across key domains relevant to AI-assisted evidence synthesis, including model characteristics, task design, benchmarking procedures, comparator definitions, evaluation metrics, reproducibility considerations, and reporting transparency.
(PDF)

S2 File Prompt Framework. Task-specific RISEN prompt framework for SLRs. Full RISEN prompts used across all GPT-5 mode variants and SLR workflow stages. Prompts can be used and adapted as reproducible templates to facilitate methodological transparency, workflow replication, and adaptation across digital psychiatry evidence synthesis contexts.
(PDF)

S2 Text Detailed description of the human-led SLR workflow. Expanded description of the human-led SLR workflow used for benchmarking, including methodological elaboration of the human researchers’ processes at each stage, reviewer expertise, consensus adjudication procedures, and blinding of human reviewers to GPT-5 outputs.
(PDF)

S3 Text Task-specific operational definitions for signal detection metrics across SLR stages. Detailed descriptions of how signal detection metrics (hits, misses, false alarms, and correct rejections) were operationalized across each SLR stage to permit benchmarking comparisons between human-led and GPT-5 workflows, with consideration of methodological differences across binary classification, structured, conceptual, and narrative outputs.
(PDF)

S1 Table Stage-specific time and personnel requirements across SLR workflows. Comparative summary of estimated time requirements and personnel involvement across each SLR task for GPT-5 Auto, Agent, and Deep Research mode variants relative to the human-led workflow.
(PDF)

S3 File Template. GRADE critical appraisal table template. Template used to guide GRADE critical appraisal procedures for human researchers and GPT-5 mode variants.
(PDF)

S2 Table Signal detection values and bounded reclassification sensitivity analyses across SLR tasks. Task-specific signal detection outcome frequencies for GPT-5 Auto, Agent, and Deep Research mode variants relative to the human-led workflow across SLR tasks. Also includes bounded reclassification sensitivity analyses for title/abstract and full-text screening under varying assumptions of human reference workflow error to contextualize performance across alternative consensus-reference scenarios.
(PDF)
