# Supplementary material for: Leveraging prompt-driven generative AI for systematic reviews in digital psychiatry: A stage-matched comparative proof-of-concept for healthcare researchers and clinicians
Source: PLOS Digit Health. 2026 Sep 10;5(9):e0001666. doi: 10.1371/journal.pdig.0001666 (PMC13561331; doi:10.1371/journal.pdig.0001666)
Supplement: Supplementary file 1 [file pdig.0001666.s001.pdf]

S1 Text: Review of existing evidence for AI in systematic review tasks

A growing body of literature has begun to consolidate knowledge on AI-assisted systematic literature reviews (SLRs), including scoping and systematic reviews [1-7], as well as narrative viewpoints [8-10] that map available tools, methodological approaches, and reported use cases across the SLR workflow. These reviews collectively demonstrate rapid expansion of AI-enabled functionalities for study identification, screening, data extraction, appraisal, and synthesis, and have been complemented by dedicated tool-focused overviews cataloguing platforms aligned to specific SLR stages. Here, we have provided here a brief overview of the AI tools currently available for SLR-related tasks (Table S1), alongside a narrative review of the current state of evidence.

Table S1.1 Evidence base for AI automation at each stage of the SLR process

Research Task	AI Tool(s)	Level of Evidence	Proof-of-Concept Status	Validation Status
Preliminary Searches of Existing Evidence	Elicit.org<br>Scispace<br>Consensus<br>Scite.AI<br>ScholarGPT<br>Sider.AI<br>Loon Lens Pro 1.0<br>Review Copilot	Low	Moderate	Emerging
Research Question Development	OpenAI<br>Elicit.org<br>SWIFT-Review<br>ReLiS<br>Nested Knowledge (RoboPICO)	Low	Early-stage	Early-stage
Eligibility Criteria Development	Elicit.org<br>OpenAI	Low	Early-stage	Limited
Search Strategy Development	Elicit.org<br>OpenAI<br>SWIFT-Review<br>ReLiS<br>Nested Knowledge (RoboPICO)	Low	Early-stage	Limited
Article Screening	OpenAI	Strong	Established	Good

	Claude<br>Rayyan<br>Abstrackr<br>SWIFT-Review<br>Colandr<br>Nested Knowledge (Robot Screener)<br>Review Copilot			
Data Extraction	Claude<br>OpenAI<br>RobotReviewer<br>Colandr<br>EPPI Reviewer 6<br>Consensus<br>SciSpace	Moderate	Emerging	Low
Article Summarization	Claude<br>OpenAI<br>RobotReviewer<br>Colandr<br>EPPI Reviewer 6<br>Consensus<br>SciSpace	Low	Early-stage	Low
Critical Appraisals and Evidence	RobotReviewer	Moderate	Emerging	Moderate

Quality Assessment	ReLIS<br>EvidenceGRADEr			
Results Synthesis	Claude<br>OpenAI<br>Consensus (Perplexity)<br>Gemini<br>Promptheus<br>ForeFront AI Chat<br>Nested Knowledge<br>EPPI-Reviewer 6	Low	Early-stage	Limited

Preliminary Searches

Several AI tools are emerging that are tailored to sub-tasks essential to carrying out preliminary searches of existing scientific evidence. In particular, Elicit.org [11, 12], Scispace [13], OpenAI [14], Consensus [15-17], Review Copilot [18], and Loon Lens Pro 1.0 [19, 20] demonstrate emerging value for rapid, high-precision preliminary searches aimed at orienting researchers to an existing evidence base, identifying influential studies, and supporting early hypothesis refinement. Comparative case studies show that these tools retrieve fewer but more topically concentrated records than traditional database searches, resulting in substantially higher precision but markedly lower sensitivity. For example, Elicit consistently under-retrieved eligible studies compared to librarian-designed searches (average sensitivity ~38–40%), yet produced far fewer irrelevant records and identified a small number of unique, otherwise missed studies, supporting its role as a complementary scoping aid rather than a standalone search method [11, 12].

It is important to recognize, however, that a growing body of commercial AI tools are

becoming available, marketed for researchers and medical science (e.g., Scite.AI, ScholarGPT, Sider.AI), but currently lack empirical evidence informing accuracy, quality, and benchmarked validation to human-centric practices [8]. Literature reviews synthesizing the evidence base emphasize AI's technical feasibility and functionality for preliminary search operations, with limited empirical validation of accuracy or reproducibility compared to human-centric practices [21-24]. This is further complemented by a comparative evaluation of eleven AI tools, in which Moens and colleagues [13] found that AI tools tended to perform poorly (compared to human researchers) for literature searches and establishing an evidence base.

Research Question Development

A handful of AI applications have been leveraged for formulating or refining research questions (e.g., OpenAI, Elicit.org, SWIFT-Review, ReLiS, and Nested Knowledge: RoboPICO), typically using a Participant, Intervention, Comparator, and Outcomes (PICO) structure [6, 8-10, 13, 25, 26]. To date, most literature available is primarily conceptual or descriptive and often used for brainstorming and structuring research aims [9, 27, 28], with minimal empirical validation benchmarked to human performance [27, 29]. Literature reviews conceptually support AI utility for assisting by efficiently identifying prior systematic reviews, surfacing domain concepts, and structuring questions using frameworks such as PICO [6, 9], but emphasize that performance is currently not sufficient to replace human researchers for research question development. This is further qualified by concerns surrounding the novelty of research question production, versus reproducing prevailing research framings, overemphasizing highly cited domains, and inadequately capturing clinical nuance or unresolved methodological controversies, limiting originality and strategic value [13]. Overall, the evidence supports positioning AI as an ideation and structuring aid for research question development, while final RQ definition remains a fundamentally human task requiring domain expertise, judgment, and critical synthesis.

Eligibility Criteria Development

Likewise, reviews of the literature conclude that current AI and LLM-based systems can assist with drafting preliminary eligibility criteria for SLRs but do not yet match human methodological performance [5, 10, 27, 30]. Commentaries and empirical demonstrations are focused on two primary tools (Elicit.org and OpenAI's ChatGPT), indicate that such tools can generate eligibility criteria that are structurally coherent and broadly aligned with PICO elements, making them potentially useful as an initial scaffold or starting point for refinement by experienced reviewers [10]. However, critical evaluations show that AI-generated criteria frequently suffer from conceptual over-inclusiveness, omission of key clinical or methodological constraints, and misalignment with the stated research question, reflecting limited domain understanding and an inability to operationalize nuanced inclusion/exclusion logic without expert oversight [14]. Large scoping reviews further demonstrate that, while AI systems are increasingly used downstream to *apply* predefined eligibility criteria during screening, far fewer studies address AI performance in *formulating* those criteria, and none provide robust human-versus-AI benchmarking for this task [5]. Taken together, the evidence supports a hybrid model in which AI may accelerate early eligibility-criteria drafting, but human expertise remains essential to ensure clinical relevance, methodological rigour, and defensibility in systematic review protocols.

Search Strategy Development

The literature consistently shows that AI tools such as Elicit.org, OpenAI, SWIFT-Review, ReLiS, and Nested Knowledge: RoboPICO can support but not replicate human expertise in search strategy development for systematic reviews [4-6, 8, 10, 31, 32]. Tool overviews and conceptual frameworks emphasize that AI can assist with early scoping, term expansion, query drafting, and translation of search strings across databases, but they also

highlight persistent risks related to insufficient recall, opaque query logic, and limited transparency, which undermine reproducibility and trust when compared with librarian- or methodologist-designed strategies [2, 33]. Empirical benchmarking studies demonstrate that AI-generated search strategies typically achieve substantially higher precision but markedly lower sensitivity than traditional human-led searches, with recall often falling below thresholds considered acceptable for systematic reviews; nevertheless, AI tools occasionally identify unique relevant studies missed by human searches, suggesting complementary rather than substitutive value [12, 13]. Case studies in biomedical reviews show that, when AI-generated search strings are closely supervised and validated by human reviewers, performance can approach human standards for specific topics, but this success is contingent on expert correction and does not generalize across domains [26]. Overall, the evidence supports a hybrid model in which AI augments search strategy development—particularly for preliminary exploration and efficiency gains—while final, protocol-defining search strategies remain a human-led task essential for ensuring completeness, methodological rigour, and defensibility.

Article Screening

The current research landscape and commercial development of AI-enabled automation for SLRs has predominantly concentrated on article screening, resulting in a comparatively robust evidence base evaluating both technical performance and validation against human reviewers [4-6, 8, 10, 31, 32]. Multiple studies have demonstrated that AI tools can deliver substantial time and workload reductions during title/abstract and full-text screening [34-36], while achieving high specificity in excluding irrelevant records [34, 37, 38], particularly when models are explicitly optimized for precision [39]. In this context, false negatives, arguably the most consequential error in screening due to the risk of erroneously excluding eligible studies [40], appear to be uncommon in several implementations; for example, Giumarra and

colleagues [34] reported a false-negative rate of only 0.3% when using Abstrackr, supporting the safety of AI-assisted screening under carefully controlled conditions.

By contrast, evidence regarding sensitivity remains mixed. Some evaluations report reduced sensitivity during title/abstract and full-text screening [34, 40], whereas others demonstrate performance approaching or matching human reviewers, particularly when tools such as Rayyan, Abstrackr, SWIFT-Review, and ChatGPT are deployed [37, 38, 41]. Variability in sensitivity appears to be context-dependent, with lower performance more frequently observed in methodologically complex reviews, those addressing multiple outcomes, or those characterized by highly imbalanced datasets. In such scenarios, AI systems may overestimate citation relevance or inconsistently apply inclusion criteria, underscoring the continued need for human oversight and validation when integrating AI into screening workflows [34].

Data Extraction

Existing studies primarily demonstrate the feasibility of AI-assisted data extraction for SLRs [1, 26, 42-44], with substantially fewer investigations providing empirical benchamarking against human performance for this task. Among studies that do report validation metrics, estimates of precision, recall, efficient, and accuracy vary widely depending on both the tool employed and the type of data extracted (i.e., sample size, population, outcome) [45-50]. Comparative evaluations indicate that overall data extraction performance is markedly higher for ELISE and OpenAI's ChatGPT (87.5% and 86.9%, respectively) than for other tools, including Humata (52%), SciSpace/Typeset (49%), and Epsilon (37.41%). An expanding body of literature further supports Claude for structured data extraction tasks [4, 46, 51-54]. However, several commercially available AI automation platforms (e.g., RobotReviewer, Colandr, EPPI Reviewer 6, Consensus) remain insufficiently validated in the peer-reviewed

literature.

Article Summaries

Studies assessing the feasibility of AI tools for generating article summaries are emerging [55-57], but remain comparatively sparse relative to evaluations of other SLR tasks. Existing investigations, primarily focused on the summarisation of randomized controlled trials and medical research content, largely emphasize feasibility and general technical capabilities [58, 59], while offering limited quantitative evaluation. Instead, most reports provide qualitative descriptions of task-specific errors and limitations, including reference hallucination, content over-generalization, and inaccurate representations of study findings [27, 60]. Notably, despite the proliferation of commercial AI tools marketed for research-grade article summarisation (e.g., Claude, OpenAI, RobotReviewer, Colandr, EPPI Reviewer 6, Consensus, SciSpace), empirical studies benchmarking performance against human summarisation remain rare [45, 58].

Quality Assessment

Critical appraisal of complex content, risk of bias assessment, and research quality evaluation are essential components to the SLR workflow that are receiving growing attention in technical development and feasibility evaluation [7, 61]. In fact, AI tools such as RobotReviewer and EvidenceGRADEr [62] are now available to support quality assessment using established frameworks such as GRADE [8, 62-64]. Emerging evidence suggests that commonly reported validation and performance metrics (e.g., precision, accuracy) are moderate to high and broadly comparable to human reviewers, particularly when risk of bias is assessed on a study-by-study basis, rather than overall quality assessment of outcomes aggregated across studies [8, 62-66]. By contrast, empirical support remains weaker for assessments of blinding and cross-study synthesis for meta-analysis [65, 66]. Although these

findings are promising, the evidence base remains preliminary; further replication and rigorous validation are required before widespread adoption can be recommended within standard medical evidence-based practice [7].

Results Synthesis

To date, a few AI tools have been proposed to support automated results synthesis for SLRs, such as OpenAI [26, 40, 51], Promptheus [24], ForeFront AI Chat, Nested Knowledge, and EPPI-Reviewer 6. However, very few studies have addressed and evaluated AI automation tools for descriptive results synthesis of demographic characteristics or study design features and/or narrative synthesis. Existing early-stage research in this domain focuses on technical AI feasibility [27, 60], with limited empirical validation [67]. Collectively, current evidence agrees that AI-automated results synthesis is feasible for first-pass supportive use in systematic and narrative reviews, but is not yet reliable as an autonomous process. Common AI platforms (e.g., Claude, OpenAI, Consensus (Perplexity), Gemini) can rapidly synthesize and compare bodies of clinical evidence and guidelines, achieving substantial time savings (35%-70%) and producing clinically intelligible summaries; however, they consistently require manual verification due to risks of reference hallucination, over-generalisation, and occasional misinterpretation of findings [27, 60]. Complementing this, ChatGPT can generate coherent, human-like narrative syntheses and section-level summaries that are difficult for clinicians to distinguish from human-written text, particularly when multi-step prompting and full-text inputs are used [27]; nevertheless, the study documents systematic weaknesses in factual accuracy, citation validity, and critical interpretation, including frequent hallucinated or erroneous references, which undermine trustworthiness for standalone results synthesis.

Current Study

Despite this expanding review and commentary landscape, the field remains notably constrained by a lack of empirical, stage-matched evaluations comparing LLM-assisted and human-led performance across multiple core SLR and research tasks, particularly within psychiatry and mental health research. Existing empirical studies tend to examine isolated tasks (e.g., screening or synthesis) or remain technically oriented, without systematically benchmarking AI and human reviewers using comparable task definitions, performance metrics, and decision thresholds across the full SLR workflow. As a result, current guidance for medical and psychiatric researchers is largely inferential rather than evidence-based, highlighting a critical need for rigorous, task-specific human-versus-LLM comparisons to inform best practices for responsible AI integration in psychiatric evidence synthesis.

Supplementary References

1. Blaizot A, Veettil SK, Saidoung P, Moreno-Garcia CF, Wiratunga N, Aceves-Martins M, et al. Using artificial intelligence methods for systematic review in health sciences: A systematic review. Research Synthesis Methods. 2022;13(3):353-62. doi: https://doi.org/10.1002/jrsm.1553.
2. Fabiano N, Gupta A, Bhambra N, Luu B, Wong S, Maaz M, et al. How to optimize the systematic review process using AI tools. JCPP Adv. 2024;4(2):e12234. Epub 20240423. doi: 10.1002/jcv2.12234. PubMed PMID: 38827982; PubMed Central PMCID: PMCPMC11143948.
3. Khalil H, Ameen D, Zarnegar A. Tools to support the automation of systematic reviews: a scoping review. Journal of Clinical Epidemiology. 2022;144:22-42. doi: https://doi.org/10.1016/j.jclinepi.2021.12.005.
4. Mikriukov A, Senokosov A, Succi G, Tormasov A, Plaksin Y, Trofimova E, et al. AI tools for automating systematic literature reviews. Proceedings of the 2025 International Conference on Software Engineering and Computer Applications2025. p. 25-30.
5. Santos ÁOd, da Silva ES, Couto LM, Reis GVL, Belo VS. The use of artificial intelligence for automating or semi-automating biomedical literature analyses: A scoping review. Journal of Biomedical Informatics. 2023;142:104389. doi: https://doi.org/10.1016/j.jbi.2023.104389.
6. Schmidt L, Sinyor M, Webb RT, Marshall C, Knipe D, Eyles EC, et al. A narrative review of recent tools and innovations toward automating living systematic reviews and evidence syntheses. Zeitschrift für Evidenz, Fortbildung und Qualität im Gesundheitswesen. 2023;181:65-75. doi: https://doi.org/10.1016/j.zefq.2023.06.007.
7. Tóth B, Berek L, Gulácsi L, Péntek M, Zrubka Z. Automation of systematic reviews of biomedical literature: a scoping review of studies indexed in PubMed. Systematic Reviews. 2024;13(1):174. doi: 10.1186/s13643-024-02592-3.
8. Ge L, Agrawal R, Singer M, Kannapiran P, De Castro Molina JA, Teow KL, et al. Leveraging artificial intelligence to enhance systematic reviews in health research: advanced

tools and challenges. Systematic Reviews. 2024;13(1):269. doi: 10.1186/s13643-024-02682-2.
9. Luo X, Chen F, Zhu D, Wang L, Wang Z, Liu H, et al. Potential Roles of Large Language Models in the Production of Systematic Reviews and Meta-Analyses. J Med Internet Res. 2024;26:e56780. Epub 25.6.2024. doi: 10.2196/56780. PubMed PMID: 38819655.
10. Qureshi R, Shaughnessy D, Gill KAR, Robinson KA, Li T, Agai E. Are ChatGPT and large language models "the answer" to bringing us closer to systematic review automation? Systematic Reviews. 2023;12(1):72. doi: 10.1186/s13643-023-02243-z.
11. Bernard N, Sagawa Y, Jr., Bier N, Lihoreau T, Pazart L, Tannou T. Using artificial intelligence for systematic review: the example of elicit. BMC Med Res Methodol. 2025;25(1):75. Epub 20250318. doi: 10.1186/s12874-025-02528-y. PubMed PMID: 40102714; PubMed Central PMCID: PMCPMC11921719.
12. Lau O, Golder S. Comparison of Elicit AI and traditional literature searching in systematic reviews using four case studies. medRxiv. 2025. doi: 10.1101/2025.06.17.25329772.
13. Moens M, Nagels G, Wake N, Goudman L. Artificial intelligence as team member versus manual screening to conduct systematic reviews in medical sciences. iScience. 2025;28(10):113559. Epub 20250912. doi: 10.1016/j.isci.2025.113559. PubMed PMID: 41079628; PubMed Central PMCID: PMCPMC12513305.
14. Guimarães NS, Joviano-Santos JV, Reis MG, Chaves RRM, Observatory of Epidemiology NHR. Development of search strategies for systematic reviews in health using ChatGPT: a critical analysis. Journal of Translational Medicine. 2024;22(1):1. doi: 10.1186/s12967-023-04371-5.
15. Apata OE, Kwok OM, Lee YH. The use of generative artificial intelligence (AI) in academic research: A review of the Consensus app. Cureus. 2025;17(7):e87297. Epub 20250704. doi: 10.7759/cureus.87297. PubMed PMID: 40755655; PubMed Central PMCID: PMCPMC12318603.
16. Faix A. Consensus: Using AI to analyze scientific literature. Library Trends. 2025;73(3):344-54. doi: 10.1353/lib.2025.a961198.
17. Tosi D. Comparing generative AI literature reviews versus human-led systematic literature reviews: A case study on big data research. IEEE Access. 2025;13:56210-9. doi: 10.1109/access.2025.3554504.
18. Zhan* J, Suvada* K, Xu M, Tian W, Cara KC, Wallace TC, et al. Accelerating the pace and accuracy of systematic reviews using AI: A validation study. medRxiv. 2024. doi: https://doi.org/10.1101/2024.12.10.24318803.
19. Janoudi G, Uzun M, Jurdana M, Fuzul E, Ivkovic J. Loon Lens 1.0 Validation: Agentic AI for Title and Abstract Screening in Systematic Literature Reviews. medRxiv. 2024:2024.09.06.24313186. doi: 10.1101/2024.09.06.24313186.
20. Janoudi G, Uzun M, Jurdana M, Hutton B. Evaluating Loon Lens Pro™, an AI-Driven Tool for Full-Text Screening in Systematic Reviews: A Validation Study. medRxiv. 2025:2025.02.11.25322087. doi: 10.1101/2025.02.11.25322087.
21. Marshall IJ, Trikalinos TA, Soboczenski F, Yun HS, Kell G, Marshall R, et al. In a pilot study, automated real-time systematic review updates were feasible, accurate, and work-saving. Journal of Clinical Epidemiology. 2023;153:26-33. doi: https://doi.org/10.1016/j.jclinepi.2022.08.013.
22. Sami AM, Rasheed Z, Kemell K-K, Waseem M, Kilamo T, Saari M, et al. System for systematic literature review using multiple AI agents: Concept and an empirical evaluation. arXiv. 2025.
23. Staudinger M, Kusa W, Piroi F, Lipani A, Hanbury A. A reproducibility and generalizability study of large language models for query generation. Proceedings of the 2024

Annual International ACM SIGIR Conference on Research and Development in Information Retrieval in the Asia Pacific Region2024. p. 186-96.
24. Torres JPF, Mulligan C, Jorge J, Moreira C. PROMPTHEUS: A human-centred pipeline to streamline SLRs with LLMs. arXiv. 2024.
25. Bigendako B, Syriani E, editors. Modeling a tool for conducting systematic reviews iteratively. 6th International Conference on Model-Driven Engineering and Software Development (MODELSWARD); 2018; Madeira, Portugal.
26. Teperikidis L, Karakasis P, Patoulias D, Boulmpou A, Kouzoukas D. Validating ChatGPT's role in systematic reviews and meta-analyses: A case study on GLP-1 receptor agonists and all-cause mortality. European Journal of Internal Medicine. 2025;134:145-7. doi: https://doi.org/10.1016/j.ejim.2024.10.017.
27. Manley K, Salingaros S, Fuchsman AC, Dong X, Spector JA. Using ChatGPT to write a literature review on autologous fat grafting. Journal of Plastic, Reconstructive & Aesthetic Surgery. 2025;105:292-304. doi: https://doi.org/10.1016/j.bjps.2025.04.015.
28. Robinson K, Bontekoe K, Muellenbach J. Integrating PICO principles into generative artificial intelligence prompt engineering to enhance information retrieval for medical librarians. J Med Libr Assoc. 2025;113(2):184-8. doi: 10.5195/jmla.2025.2022. PubMed PMID: 40342302; PubMed Central PMCID: PMCPMC12058339.
29. Gosak L, Štiglic G, Pruinelli L, Vrbnjak D. PICOT questions and search strategies formulation: A novel approach using artificial intelligence automation. Journal of Nursing Scholarship. 2025;57(1):5-16. doi: https://doi.org/10.1111/jnu.13036.
30. Topol EJ. High-performance medicine: the convergence of human and artificial intelligence. Nature Medicine. 2019;25(1):44-56. doi: 10.1038/s41591-018-0300-7.
31. Syriani E, David I, Kumar G. Screening articles for systematic reviews with ChatGPT. Journal of Computer Languages. 2024;80:101287. doi: https://doi.org/10.1016/j.cola.2024.101287.
32. Tomczyk P, Brüggemann P, Vrontis D. AI meets academia: Transforming systematic literature reviews. EuroMed Journal of Business. 2024. doi: https://doi.org/10.1108/EMJB-03-2024-0055.
33. MacFarlane A, Russell-Rose T, Shokraneh F. Search strategy formulation for systematic reviews: Issues, challenges and opportunities. Intelligent Systems with Applications. 2022;15:200091. doi: https://doi.org/10.1016/j.iswa.2022.200091.
34. Giummarra MJ, Lau G, Gabbe BJ. Evaluation of text mining to reduce screening workload for injury-focused systematic reviews. Injury Prevention. 2020;26(1):55-60. doi: 10.1136/injuryprev-2019-043247.
35. van de Schoot R, de Bruin J, Schram R, Zahedi P, de Boer J, Weijdema F, et al. An open source machine learning framework for efficient and transparent systematic reviews. Nature Machine Intelligence. 2021;3(2):125-33. doi: 10.1038/s42256-020-00287-7.
36. Abogunrin S, Muir JM, Zerbini C, Sarri G. How much can we save by applying artificial intelligence in evidence synthesis? Results from a pragmatic review to quantify workload efficiencies and cost savings. Frontiers in Pharmacology. 2025;Volume 16 - 2025. doi: 10.3389/fphar.2025.1454245.
37. Li J, Kabouji J, Bouhadoun S, Tanveer S, Filion KB, Gore G, et al. Sensitivity and specificity of alternative screening methods for systematic reviews using text mining tools. Journal of Clinical Epidemiology. 2023;162:72-80. doi: https://doi.org/10.1016/j.jclinepi.2023.07.010.
38. Wilson E, Cruz F, Maclean D, Ghanawi J, McCann Sarah K, Brennan Paul M, et al. Screening for in vitro systematic reviews: a comparison of screening methods and training of a machine learning classifier. Clinical Science. 2023;137(2):181-93. doi: 10.1042/cs20220594.

39. Feng Y, Liang S, Zhang Y, Chen S, Wang Q, Huang T, et al. Automated medical literature screening using artificial intelligence: a systematic review and meta-analysis. Journal of the American Medical Informatics Association. 2022;29(8):1425-32. doi: 10.1093/jamia/ocac066.
40. Khraisha Q, Put S, Kappenberg J, Warraitch A, Hadfield K. Can large language models replace humans in systematic reviews? Evaluating GPT-4's efficacy in screening and extracting data from peer-reviewed and grey literature in multiple languages. Research Synthesis Methods. 2024;15(4):616-26. doi: https://doi.org/10.1002/jrsm.1715.
41. Li M, Sun J, Tan X. Evaluating the effectiveness of large language models in abstract screening: a comparative analysis. Systematic Reviews. 2024;13(1):219. doi: 10.1186/s13643-024-02609-x.
42. Hill JE, Harris C, Clegg A. Methods for using Bing's AI-powered search engine for data extraction for a systematic review. Research Synthesis Methods. 2024;15(2):347-53. doi: https://doi.org/10.1002/jrsm.1689.
43. Lagisz M, Mizuno A, Morrison K, Pollo P, Ricolfi L, Yang Y, et al. Using Elicit AI research assistant for data extraction in systematic reviews: a feasibility study across environmental and life sciences. EcoEvoRxiv. 2025.
44. Reason T, Langham J, Gimblett A. Automated Mass Extraction of Over 680,000 PICOs from Clinical Study Abstracts Using Generative AI: A Proof-of-Concept Study. Pharmaceutical Medicine. 2024;38(5):365-72. doi: 10.1007/s40290-024-00539-6.
45. Bui DDA, Del Fiol G, Hurdle JF, Jonnalagadda S. Extractive text summarization system to aid data extraction from full text in systematic review development. Journal of Biomedical Informatics. 2016;64:265-72. doi: https://doi.org/10.1016/j.jbi.2016.10.014.
46. Gartlehner G, Kahwati L, Hilscher R, Thomas I, Kugley S, Crotty K, et al. Data extraction for evidence synthesis using a large language model: A proof-of-concept study. Res Synth Methods. 2024;15(4):576-89. Epub 20240303. doi: 10.1002/jrsm.1710. PubMed PMID: 38432227.
47. Gobin M, Gosnat M, Toure S, Faik L, Belafa J, Villedieu de Torcy A, et al. From data extraction to analysis: a comparative study of ELISE capabilities in scientific literature. Frontiers in Artificial Intelligence. 2025;Volume 8 - 2025. doi: 10.3389/frai.2025.1587244.
48. Meliante LA, Coco G, Rabiolo A, De Cillà S, Manni G. Evaluation of AI Tools Versus the PRISMA Method for Literature Search, Data Extraction, and Study Composition in Glaucoma Systematic Reviews: Content Analysis. JMIR AI. 2025;4:e68592. Epub 5.9.2025. doi: 10.2196/68592.
49. Mitchell E, Are EB, Colijn C, Earn DJ. Using artificial intelligence tools to automate data extraction for living evidence syntheses. PloS one. 2025;20(4):e0320151.
50. Rokhshad R, Mohammad FD, Nomani M, Mohammad-Rahimi H, Schwendicke F. Chatbots for conducting systematic reviews in pediatric dentistry. Journal of Dentistry. 2025;158:105733. doi: https://doi.org/10.1016/j.jdent.2025.105733.
51. Insuk S, Boonpattharatthiti K, Booncharoen C, Chaipitak P, Rashid M, Veettil SK, et al. How well do ChatGPT and Claude perform in study selection for systematic review in obstetrics. J Med Syst. 2025;49(1):110. Epub 20250904. doi: 10.1007/s10916-025-02246-4. PubMed PMID: 40906005.
52. Cuthbert C, Twomey R, Bansal M, Rana B, Dhruva T, Livingston V, et al. The role of exercise for pain management in adults living with and beyond cancer: a systematic review and meta-analysis. Support Care Cancer. 2023;31(5):254. Epub 20230411. doi: 10.1007/s00520-023-07716-4. PubMed PMID: 37039883; PubMed Central PMCID: PMCPMC10088810.
53. Kasireddy E, Chow C, Collet J, Pourrahmat MM, Fazeli MS. Evaluating the performance of Claude 3.7 Sonnet in data extraction automation for systematic literature

reviews. Value Health Reg Issues. 2025;53:101539. Epub 20251111. doi: 10.1016/j.vhri.2025.101539. PubMed PMID: 41223819.
54. Konet A, Thomas I, Gartlehner G, Kahwati L, Hilscher R, Kugley S, et al. Performance of two large language models for data extraction in evidence synthesis. Res Synth Methods. 2024;15(5):818-24. Epub 20240619. doi: 10.1002/jrsm.1732. PubMed PMID: 38895747.
55. Atkinson CF. Cheap, quick, and rigorous: Artificial intelligence and the systematic literature review. Social Science Computer Review. 2024;42(2):376-93. doi: 10.1177/08944393231196281.
56. Bilal IM, Fang Z, Arana-Catania M, van Lier F-A, Valarde JO, Bregazzi H, et al. Mcahine learning information retrieval and summarisation to support SLR on outcomes about contracting. arXiv. 2024:1-31.
57. Grbin L, Nichols P, Russell F, Fuller-Tyszkiewicz M, Olsson CA. The development of a living knowledge system and implications for future systematic searching. Journal of the Australian Library and Information Association. 2022;71(3):275-92.
58. Li J, Deng Y, Sun Q, Zhu J, Tian Y, Li J, et al. Benchmarking large language models in evidence-based medicine. IEEE J Biomed Health Inform. 2025;29(9):6143-56. doi: 10.1109/JBHI.2024.3483816. PubMed PMID: 39437276.
59. Afzal M, Alam F, Malik KM, Malik GM. Clinical context-aware biomedical text summarization using deep neural network: model development and validation. J Med Internet Res. 2020;22(10):e19810. Epub 20201023. doi: 10.2196/19810. PubMed PMID: 33095174; PubMed Central PMCID: PMCPMC7647812.
60. Ayala-Yáñez R, Grünebaum A, Chervenak FA. Integrating generative AI in perinatology: applications for literature review. Journal of Perinatal Medicine. 2025. doi: doi:10.1515/jpm-2025-0392.
61. Schmidt L, Cree I, Campbell F, group WEM. Digital Tools to Support the Systematic Review Process: An Introduction. Journal of Evaluation in Clinical Practice. 2025;31(3):e70100. doi: https://doi.org/10.1111/jep.70100.
62. Šuster S, Baldwin T, Lau JH, Jimeno Yepes A, Martinez Iraola D, Otmakhova Y, et al. Automating Quality Assessment of Medical Evidence in Systematic Reviews: Model Development and Validation Study. J Med Internet Res. 2023;25:e35568. Epub 13.3.2023. doi: 10.2196/35568. PubMed PMID: 36722350.
63. Marshall IJ, Wallace BC. Toward systematic review automation: a practical guide to using machine learning tools in research synthesis. Systematic Reviews. 2019;8(1):163. doi: 10.1186/s13643-019-1074-9.
64. Millard LA, Flach PA, Higgins JP. Machine learning to assist risk-of-bias assessments in systematic reviews. International Journal of Epidemiology. 2015;45(1):266-77. doi: 10.1093/ije/dyv306.
65. Hirt J, Meichlinger J, Schumacher P, Mueller G. Agreement in Risk of Bias Assessment Between RobotReviewer and Human Reviewers: An Evaluation Study on Randomised Controlled Trials in Nursing-Related Cochrane Reviews. Journal of Nursing Scholarship. 2021;53(2):246-54. doi: https://doi.org/10.1111/jnu.12628.
66. Jardim PSJ, Rose CJ, Ames HM, Echavez JFM, Van de Velde S, Muller AE. Automating risk of bias assessment in systematic reviews: a real-time mixed methods comparison of human researchers to a machine learning system. BMC Medical Research Methodology. 2022;22(1):167. doi: 10.1186/s12874-022-01649-y.
67. Tang L, Sun Z, Idnay B, Nestor JG, Soroush A, Elias PA, et al. Evaluating large language models on medical evidence summarization. npj Digital Medicine. 2023;6(1):158. doi: 10.1038/s41746-023-00896-7.
