# Supplementary material for: Leveraging prompt-driven generative AI for systematic reviews in digital psychiatry: A stage-matched comparative proof-of-concept for healthcare researchers and clinicians
Source: PLOS Digit Health. 2026 Sep 10;5(9):e0001666. doi: 10.1371/journal.pdig.0001666 (PMC13561331; doi:10.1371/journal.pdig.0001666)
Supplement: Supplementary file 2 [file pdig.0001666.s002.pdf]

S1 Checklist: Design and Evaluation of AI in Literature Reviews – Benchmarking (DEAL-B) checklist for standardized reporting

Section / Topic	No.	Item	Information	Page #	No / N/A
Model Information & Study Setup	1.1	LLM Model Information	Provide name(s), version(s), and dates of use. Indicate if using default or modified version(s).	GPT-5 Auto, Agent, & Deep Research; October 2025<br>pp. 12, 15	
	1.2	Model Selection & Usage Purpose	Briefly explain the rationale for choosing the specific LLM and its intended application (e.g., content generation, classification).	Evidence-based (emerging) across SLR tasks and ease of use for clinicians and non-technical researchers<br>pp. 9-11, 13-15	
	1.3	Parameter Adjustments	List basic parameter adjustments (e.g., temperature, max tokens, penalties). State if using default settings.	Default settings<br>p. 15	N/A
	1.4	API / Tool Information	Specify the API or platform (e.g., OpenAI API, Hugging Face) used to interact with the LLM.	Open AI<br>p. 15	

	1.5	Custom Instructions	Describe any specific instructions or system prompts given to the model (e.g., 'You are a helpful assistant…').	Supplementary Materials 3	
	1.6	Most Recent Training Date	Provide the most recent training date of the LLM model used.	October 2025<br>p. 15	
	1.7	Annotation Process	Outline any annotation processes, including team roles, guidelines, and quality control measures.	Procedures: prompt generation and workflow execution, mixed methods analytic strategy<br>pp. 13-15	
	1.8	Compute Resources	Specify compute resources (e.g., GPU/CPU hours, cost) used in model usage or fine-tuning.	Time and cost<br>pp. 22-23 & Supplementary Materials 6	
	1.9	Ethical Considerations	Indicate Institutional Review Board (IRB) or ethics committee approvals, and patient consent/waivers, as applicable.		N/A
	1.10	Funding & Disclosures	Disclose funding sources and any	Competing interests	

			potential conflicts of interest (COI).	& funding statement<br>p. 54	
Prompt Engineering	2.1	Prompting Strategies	Describe the key prompts tested or used, including techniques such as zero-shot, few-shot, or chain-of-thought (CoT) prompting.	RISEN framework<br>pp. 13-14<br>Supplementary Materials 3	
	2.2	Iterative Prompting	Describe any iterative process used to refine prompts based on model outputs.		N/A
	2.3	Example Prompts	Provide examples of the prompts that were most effective. Optionally include examples as supplemental material.	Supplementary Materials 3	
	2.4	Multi-Step Prompting	If multiple stages of prompts were used (e.g., multi-agent scenarios), describe the sequence and rationale.		N/A
Output Evaluation	3.1	Basic Evaluation Methods	Describe how outputs were evaluated (e.g., human evaluation, automated quality checks).	Mixed methods analytic strategy<br>pp. 16-22	

	3.2	Metrics for Evaluation	List any metrics used to assess the outputs (e.g., coherence, fluency, relevance). If no formal metrics, describe qualitative observations.	Time, accuracy, specificity, sensitivity, inter-rater agreement, compliance, word limits, summary quality, qualitative observations<br><br>pp. 16-22	
	3.3	Post-Processing of Outputs	Indicate if any post-processing (e.g., filtering, cleaning, or human review) was applied to improve the final output.	None, evaluating raw outputs	N/A
Bias & Fairness	4.1	Basic Bias Detection	Provide general approach to identifying bias, including qualitative observations of fairness issues in model outputs.	Human researchers blind to GPT outputs, qualitative observations of GPT outputs blind to human outputs<br><br>pp. 13, 16, 21-22	
	4.2	Synthetic Data Bias	For synthetic data, describe basic checks to ensure fairness and data quality.		N/A

	4.3	Fairness Observations	Note subjective observations of bias, including cases where model may exhibit consistent bias in responses.		N/A
Model Stochasticity	5.1	Basic Consistency Check	Describe steps taken for multiple queries to ensure consistency, including general observations of response variability.		N/A
	5.2	Qualitative Stochasticity Report	Summarize any observed response variability, and outline methods used to maintain reliability even without quantitative metrics.	Additional prompting requirements or failure to execute<br>pp. 15, 28-29	
