# Supplementary material for: Leveraging prompt-driven generative AI for systematic reviews in digital psychiatry: A stage-matched comparative proof-of-concept for healthcare researchers and clinicians
Source: PLOS Digit Health. 2026 Sep 10;5(9):e0001666. doi: 10.1371/journal.pdig.0001666 (PMC13561331; doi:10.1371/journal.pdig.0001666)
Supplement: Supplementary file 3 [file pdig.0001666.s003.pdf]

S1 Prompt Framework: RISEN prompt framework for SLRs

General - Training

Role: You are a health sciences researcher working as part of a team to conduct a systematic literature review.

Instruction: You are being trained on how to complete systematic reviews, using the Gernstein guidelines for systematic reviews from the University of Toronto (available at https://guides.library.utoronto.ca/systematicreviews), the on-demand workshops for literature reviews available through Unity Health Toronto (available at https://guides.hsict.library.utoronto.ca/c.php?g=740056&p=5369889), and the GRADE framework for quality assessments of heterogeneous study designs (available at https://gdt.gradepro.org/app/handbook/handbook.html).

Steps: Review each training resource to familiarize yourself with the systematic review and critical appraisal process.

Goal: The ultimate goal of this task is to train you on the gold-standard methods used for systematic literature reviews for medical research so you can conduct a review in the domain of digital mental health.

Narrowing: Focus training on the resources provided here, so you receive the same training as other members of the team. You do not need to review content from other websites or sources.

You will have 8 tasks to work through to accomplish this project: 1) preliminary search of existing reviews and meta-analyses in the field, 2) innovative research question development, 3) search strategy development, 4) eligibility criteria development, 5) article screening, 6) data extraction, 7a) article summarization, 7b) critical appraisal and quality assessment, and 8) results synthesis. What is required for each task will be specified in order. You should provide output at each stage and should not proceed to the next task until you receive the next prompt.

Task 1: Preliminary Searches

Role: You are a health sciences researcher on a team conducting the first task in your systematic literature review: preliminary searches of existing reviews and meta-analyses.

Instruction: Search the PROSPERO (available here: https://www.crd.york.ac.uk/PROSPERO/search), PubMed (available here:), arXiv (available here:), and medarXiv (available here:) database using the keywords “virtual reality” AND “exergaming” AND “depression”. Generate a list of relevant reviews and meta-analyses underway (PROSPERO) or published (PubMed, arXiv, or medarXiv) relevant to virtual reality exergaming as a potential treatment for depression.

Steps: Start by searching the PROSPERO database for registered reviews and meta-analyses relevant to virtual reality exergaming as a potential treatment for depression. Create a list of all relevant reviews and meta-analyses with the registration date and project status. Next, search arXiv and medarXiv for literature reviews or meta-analyses relevant to virtual reality exergaming as a potential treatment for depression. Create a list of all relevant reviews and meta-analyses with publication date and peer-review status (if available). Finally, search PubMed for published peer-reviewed reviews and meta-analyses relevant to virtual reality exergaming in depression. Create a list of all relevant articles with publication date and journal.

End Goal: In the end, provide a concise list of all relevant reviews and meta-analyses (ongoing, under review, and published) investigating the feasibility and treatment potential of virtual reality exergaming for depression. For each review/meta-analysis listed, provide a brief overview of study characteristics (e.g., study design(s), number of studies included, project status, year of publication, type of review/meta-analysis). Overall, ensure a general and concise interpretation of the results (i.e., has this topic been reviewed to saturation?, are there gaps in the literature that remain to be informed?) and discuss any limitations that may challenge conducting reviews or meta-analyses on this topic.

Narrowing: Limit search results to reviews relevant to the intersection of 1) depression (not other mental health conditions), 2) virtual reality (not other digital intervention modalities), and 3) exergaming (not other therapeutic modalities). Do not include reviews or meta-analyses only investigating 1 or 2 of these components (depression, virtual reality, and exergaming) or reviews in other medical conditions. Do not provide an exhaustive list of findings; instead, provide a concise summary table of relevant search results and assess the volume of potentially relevant studies.

Task 2: Research Question Development

Role: You are a health sciences researcher on a team conducting the second task in your systematic literature review: research question development.

Instruction: Based on your preliminary search of the literature (Task 1), generate novel, innovative research questions for a systematic literature review and/or meta-analysis to inform the potential of virtual reality exergaming for depression.

Steps: First, identify gaps in the literature that can be addressed/answered using systematic literature review and/or meta-analysis methodology. Next, carefully define the gaps and unanswered questions in the literature - including relevant outcomes, definitions, and concepts to consider. Then generate proposed research questions.

End Goal: In the end, provide me with novel, innovative research questions (that have not been done before) that can be used for systematic reviews and/or meta-analyses addressing outstanding topics in the domain of virtual reality exergaming in depression.

Narrowing: Propose your best research question ideas; do not provide an exhaustive list. Instead, provide a concise list limited to research questions that can be answered/addressed using systematic literature review and/or meta-analysis methods. Research questions must be relevant to the intersection of 1) depression (not other mental health conditions), 2) virtual reality (not other digital intervention modalities), and 3) exergaming (not other therapeutic modalities). Do not include research questions that only investigate 1 or 2 of these components (depression, virtual reality, and exergaming), only focus on other medical conditions, or which cannot be answered using systematic literature review or meta-analysis methods.

Task 3: Eligibility Criteria Development

Role: You are a health sciences researcher on a team conducting the third task in your systematic literature review: development of article eligibility criteria.

Instruction: Generate a list of inclusion and exclusion criteria for scientific articles to be included in your systematic review investigating the feasibility and treatment potential of immersive virtual reality exergaming for depression. This review does not have restrictions on study design, depression measures, or comparators. Full text articles must be available in English, and year of publication limits may be set as appropriate.

Steps: First, identify key inclusion criteria (PICO: participants, intervention, comparator, outcomes) for scientific articles that will be included in the review. Then, specify key exclusion criteria (PICO: participants, intervention, comparator, outcomes) that would make an article ineligible for review on this topic.

End Goal: In the end, provide a list of inclusion and exclusion criteria that will determine if an article is eligible or not for a systematic review on virtual reality exergaming in depression. Organize the criteria to follow PICO format.

Narrowing: Do not provide an exhaustive list of potential criteria. Instead, provide a concise list limited to characteristics that are necessary to identify relevant research studies investigating virtual reality exergaming for depression from those that do not. Eligibility criteria must capture the intersection of 1) depression (not other mental health conditions), 2) virtual reality (not other digital intervention modalities), and 3) exergaming (not other therapeutic modalities). Do not include criteria that will only investigate 1 or 2 of these components (depression, virtual reality, and exergaming), only focus on other medical conditions, or which are too narrow (restrictive) to capture meaningful results.

Task 4: Search Strategy Development

Role: You are a health sciences researcher on a team conducting the fourth task in your systematic literature review: development of the review search strategy.

Instruction: Prepare a systematic review search strategy (with keywords and MeSH terms) relevant to virtual reality exergaming in depression for MEDLINE (https://www.nlm.nih.gov/medline/medline_home.html), Embase (https://www.embase.com/landing?status=grey), and PsycINFO (https://www.apa.org/pubs/databases/psycinfo) scientific/medical databases. The search strategy should follow PRISMA-S structure (https://systematicreviewsjournal.biomedcentral.com/articles/10.1186/s13643-020-01542-z).

Steps: First, identify keywords and MeSH terms relevant to the intersection of 1) depression, 2) virtual reality, and 3) exergaming. Next, prepare a PRISMA-S search strategy for each article database (MEDLINE, Embase, and PsycINFO) that will locate terms anywhere in the manuscript (not just title or abstract).

End Goal: In the end, provide PRISMA-S search strategies that can be used in each article database to locate potentially relevant articles for the review. Keywords should search the whole manuscript, not just the title and/or abstract.

Narrowing: Do not provide an exhaustive list of keywords with alternate spellings. Instead use truncation conventions (e.g., *, $) to capture nuances. Keywords and MeSH terms should ultimately culminate at the intersection of 1) depression (not other mental health conditions), 2) virtual reality (not other digital intervention modalities), and 3) exergaming (not other therapeutic

modalities). Do not include a search strategy that will only investigate 1 or 2 of these components (depression, virtual reality, and exergaming), only focuses on other medical conditions, or which include irrelevant terms.

Task 5: Article Screening

Role: You are a health sciences researcher on a team conducting the fifth task in your systematic literature review: article screening.

Instruction: Go row-by-row through the List of Articles Identified and determine if each article is eligible (Yes), excluded (No), or undetermined (Maybe) for this review using the attached Eligibility Criteria. If the article meets all inclusion criteria and no exclusion criteria, mark as "Yes" (eligible). If the article meets any exclusion criteria, mark as "No" (excluded). If you are not sure, mark as "Maybe" (underdetermined).

Steps: First, create 3 new columns for the Excel sheet: "Title/Abstract Eligibility Decision", "Full Text Eligibility Decision" and "Reason for Exclusion". Next, review the relevancy of each article in the list (row-by-row) based exclusively on the Title and Abstract using the attached Eligibility Criteria. Determine if the article is eligible ("Yes"), excluded ("No"), or undetermined ("Maybe") and indicate your eligibility decision (yes, no, maybe) in the respective row of the "Title/Abstract Eligibility Decision" column. Once all article titles/abstracts have been screened, review the full text of each article marked as "Yes" or "Maybe" against the Eligibility Criteria. Determine if the article full text is eligible ("Yes") or excluded ("No") and indicate your eligibility decision (yes, no) in the respective row of the "Full Text Eligibility Decision" column. If excluded, determine the primary reason for exclusion (based on the attached Reasons for Exclusion document) and indicate this in the respective row of the "Reason for Exclusion" column. If you cannot access the full text, the article should be excluded and marked as "Cannot access" (eligibility reason). After you have finished reviewing all articles for eligibility, generate a PRISMA flow diagram and summarize the number of articles eligible/ineligible at each stage.

End Goal: In the end, have a concise list of eligible articles to be included in the systematic literature review and a separate list of ineligible articles with reasons for exclusion. You should also have a PRISMA flow diagram that shows how many (and why) articles were included/excluded at each stage of screening (import, duplicates, title/abstract screening, full text screening).

Narrowing: Ensure you only screen full text eligibility for articles that have passed ("Yes" or "Maybe") title/abstract screening; do not conduct full text screening on articles marked as excluded ("No") at title/abstract screening. Reasons for exclusion should be limited to the list provided.

Task 6: Data Extraction

Role: You are a health sciences researcher on a team conducting the sixth task in your systematic literature review: data extraction.

Instruction: For each of the attached articles, extract the following information into the attached Dataset Extraction Template (.xlsx file) with one row per article. If any outcome variable is not reported or available in the full text, mark as “N/A” (not applicable).

Steps: First, create a new row for each article provided. Start with the first article uploaded and extract all variables/outcomes listed in the dataset template. Enter the extracted data into the .csv file as you go. Once complete, repeat these steps for each article provided.

End Goal: In the end, you will have a complete dataset (.csv) with extracted outcomes for all articles.

Narrowing: When providing quantitative values, round to 2 decimal places. When providing qualitative values or descriptions, ensure the information is concise and succinct yet informative. Ensure you only include the specified outcomes in the dataset, do not add new variables or outcomes not specified here.

Task 7a: Article Summaries

Role: You are a health sciences researcher on a team conducting the seventh task in your systematic literature review: article summarization.

Instruction: For each of the attached articles, summarize the main (unique) methods, results, and conclusions relevant to virtual reality exergaming in depression in 250-500 words. Summaries should be generated based on your understanding of the full text content, providing an overview of the main takeaways of the article and what the study contributes to the scientific/medical understanding of virtual reality exergaming for depression.

Steps: Start with the first article attached. Read the article and then write a 250-500-word summary of the key methods, results, and conclusions. Then, repeat this process for all articles provided.

End Goal: In the end, you will have an interpretive summary for each article included in the review.

Narrowing: Summaries should be generative content, not plagiarized or taken verbatim from the article itself, and should extend beyond what is included in the abstract. Ensure that summaries are

restricted to conclusions made by the authors from the primary study data and not from previous research studies or your own conclusions. Summarization of the background literature, rationale, and cited sources should not be included. Instead, summaries should focus on key objectives, methodological approaches, results, and/or conclusions relevant to virtual reality exergaming in depression. Objectives, methods, results, and/or conclusions not relevant to the intersection of virtual reality exergaming for depression should not be summarized here.

Task 7b: Critical Appraisals

Role: You are a health sciences researcher on a team conducting the seventh task in your systematic literature review: critical appraisals of included studies.

Instruction: For each of the attached articles, critically appraise the quality and trustworthiness of the evidence using the GRADE framework using the attached GRADE Appraisal Table. Evaluate each component of the GRADE framework - initial certainty, risk of bias, inconsistency, indirectness, imprecision, publication bias, upgrading factors (non-RCT only), and overall certainty (final rating) - based on the GRADE handbook criteria (https://gradepro.org/handbook). Brief justification notes should be provided for each rating.

Steps: Start with the first article attached. Read the article and complete the GRADE Appraisal Table. Repeat for each article provided. Once all articles have been appraised, generate a Summary Table of Findings.

End Goal: In the end, you will have critical appraisal tables for each article included in the review, as well as an overall summary table of findings.

Narrowing: GRADE domain ratings/assessments should be restricted to the categorical options specified (Initial Certainty: high, low; Upgrading Factors: +1, +2; Overall Certainty: high, moderate, low, very low; all other domains: 0, -1, -2), do not create new response options. Justification notes should be clear and succinct with a maximum of 3 sentences.

Step 8: Result Synthesis

Role: You are a health sciences researcher on a team conducting the ninth, and final, task in your systematic literature review: results synthesis.

Instruction: Conduct descriptive statistics to analyze and report Study & Intervention Design and Demographics characteristics across the target articles based on the attached data file. A narrative synthesis of key findings across studies should also be generated based on Article Summaries and Critical Appraisals (attached).

Steps: First, compute descriptive analyses for Study & Intervention Design and Demographics characteristics, reporting all findings in separate tables. (Next, use the provided article summaries and GRADE appraisals to determine recurring patterns and report key findings across studies.

End Goal: In the end, all target studies for this review will be analyzed, synthesized, and reported in tables and narrative summaries. The results generated here will inform the feasibility and treatment potential of virtual reality exergaming for depression, based on studies meeting our research criteria.

Narrowing: Quantitative metrics should be reported to 2 decimal places. If sufficient data is not available to compute a quantitative metric, record as "N/A" (not applicable) in the output table, Do not make up values. Narrative observations should be succinct and concise yet contain sufficient detail to be understood on their own (without referring back to the article or dataset).
