# Supplementary material for: Leveraging prompt-driven generative AI for systematic reviews in digital psychiatry: A stage-matched comparative proof-of-concept for healthcare researchers and clinicians
Source: PLOS Digit Health. 2026 Sep 10;5(9):e0001666. doi: 10.1371/journal.pdig.0001666 (PMC13561331; doi:10.1371/journal.pdig.0001666)
Supplement: Supplementary file 4 [file pdig.0001666.s004.pdf]

S2 Text: Detailed description of the human-led SLR workflow

A postdoctoral research fellow with experience conducting SLRs relevant to mental health conducted preliminary searches for published or ongoing SLRs on PROSPERO, arXiv, medarXiv, and PubMed databases in May 2024. Research questions and eligibility criteria were developed internally and recorded in researcher's notes (Microsoft Word). Search strategies were developed in consultation with a knowledge synthesis expert and executed by the first author using the Ovid platform. MEDLINE, Embase, and APA PsycINFO article databases were searched independently for all articles published between January 1st, 2000 through May 23rd, 2025. This year of publication restriction was implemented in line with comparable reviews in the VR intervention space to focus on modern immersive VR technologies. Covidence was used to screen articles (title/abstract and full-text) and facilitate data extraction and consensus, blinded to GPT-5 outputs. Human researchers then prepared article summaries and critically appraised each study (N = 10) using the Grading of Recommendations, Assessment, Development and Evaluation (GRADE) framework (Guyatt et al., 2008). This framework was selected for its ability to evaluate multiple dimensions of evidence quality (e.g., risk of bias, inconsistency, indirectness, imprecision, publication bias), inclusive of a diverse array of research designs. This makes it particularly suitable for synthesizing heterogeneous evidence and increasing generalizability and applicability to healthcare reviews beyond randomized controlled trials. Finally, a narrative synthesis of result findings (based on the article summaries, critical appraisals, and human-census datafile) was generated by the human researchers. All tasks in the human-led workflow were reviewed and consensus-approved by the first author prior to implementation of the LLM workflow; thus, the human-led workflow was executed blind to GPT-5 mode variant outputs.
