# Supplementary material for: Leveraging prompt-driven generative AI for systematic reviews in digital psychiatry: A stage-matched comparative proof-of-concept for healthcare researchers and clinicians
Source: PLOS Digit Health. 2026 Sep 10;5(9):e0001666. doi: 10.1371/journal.pdig.0001666 (PMC13561331; doi:10.1371/journal.pdig.0001666)
Supplement: Supplementary file 5 [file pdig.0001666.s005.pdf]

S3 Text: Task-specific operational definitions for signal detection metrics across SLR stages

Because the SLR tasks evaluated in this study differed substantially in output structure, methodological complexity, and evaluability, signal detection analyses required task-specific operational definitions. Binary classification tasks (e.g., article screening) permitted conventional inclusion/exclusion modelling. However, conceptual, structured, and narrative tasks (e.g., research question development, search strategy development, article summarization) required correspondence-based scoring between human consensus-reference outputs and GPT-5 generated responses. Accordingly, hits, misses, false alarms, and correct rejections were operationalized differently according to task characteristics. These metrics should therefore be interpreted as indicators of task-specific alignment with the consensus-based human workflow rather than direct measures of overall systematic review performance.

Preliminary Searches

For the preliminary scoping search, the search results generated by each GPT-5 mode variant for each database searched (PROSPERO, arXiv, medarXiv, and PubMed) were compared to the search results recorded by the human researchers. Relevant reviews (registered, preprints, or published) identified by both the human researcher and the GPT-5 mode variant were marked as a hit. Relevant reviews identified by the human researcher not included in the GPT-5 output were marked as a miss. Reviews deemed irrelevant by the human researcher (e.g., not exergaming, no depression measures/considerations) but included in the GPT-5 output were marked as a false alarm; however, if the GPT-5 output further specified the irrelevance of the article (and was instead mentioning it for completeness of process), then the item was marked as a correct rejection.

Research Question Development

For proposed research questions, the list of relevant research questions generated by the GPT-5 variant was compared item-by-item to the list of research questions generated by the first author before commencing the project (blind to GPT-5 outputs). If a research question topic (e.g., feasibility, treatment potential) was proposed by the human researcher and LLM, the item was marked as a hit. Notably, to permit variability in generation style between humans and LLM mode variants, the precise wording or phrasing of research questions was not necessary to achieve hit status (i.e., topic gist was sufficient). Research questions posed by the human researcher but not by the GPT-5 mode variant were marked as misses, whereas questions/topics generated by the LLM variants but not considered by human researchers were marked as false alarms. In this case, false alarms are not necessarily erroneous – instead, they may reflect new ideas not previously considered by human researchers. Correct rejections (research questions not posed by the human researcher or LLM) were not possible to quantify.

Eligibility Criteria Development

The inclusion and exclusion criteria developed by the first author in consultation with a knowledge synthesis expert were compared item-by-item to the eligibility criteria generated by each GPT-5 mode variant. Article inclusion criteria (e.g., adults, depression measures, immersive VR) specified by the human researcher and the LLM were marked as hits. In contrast, exclusionary criteria (e.g., no English full-text, grey literature) identified in both human and LLM outputs were marked as correct rejections. Criteria identified by the human researcher but not included in the LLM output were marked as misses, and instances where GPT-5 mode variants proposed criteria not included by the researcher were marked as false alarms.

Search Strategy Development

The PRISMA-S structured search strategies (MEDLINE, Embase, and APA PsycINFO) developed by the research team (in consultation with a knowledge synthesis expert) were compared term-by-term (MeSH term or keyword string) to the structured search strategies generated by each GPT-5 mode variant. Because of stylistic differences in keyword string approach (e.g., the research team used truncation conventions, such as asterisks, and broadened keyword searches to the full-text manuscript, whereas GPT-5 mode variants generated full-word terms that were searched in the standard title, abstract, and keyword fields), approximations (e.g., exergaming/exergames vs. exergam*) sufficed as the items for comparison. If a search term was captured in the human researchers' and GPT mode variant's strategy (regardless of which line it was presented on), the item was marked as a hit. MeSH and keyword search terms specified by human researchers but not captured in the GPT-5 mode variant output were marked as misses, whereas terms included by the LLM but not by the research team were marked as false alarms. In this case, it was not possible to quantify correct rejections (terms not included by the human researchers or LLM).

Article Screening

GPT-5 mode variants' performance was assessed relative to human researchers' screening decisions at both stages of article screening (title/abstract and full-text) by comparing eligibility decisions for each article. If an article was deemed relevant by human researchers and the GPT-5 mode variant, it was marked as a hit. Relevant articles (included by human researchers) that were marked for exclusion in a GPT-5 output were marked as misses. Articles deemed irrelevant (and thus excluded from the review) by the research team and LLM were marked as correct rejections; irrelevant articles rejected by human researchers but included in GPT-5 outputs were marked as false alarms.

Data Extraction

Data entries generated by the GPT-5 mode variants were qualitatively compared cell-by-cell with the consensus-approved data file extracted by the research team. Descriptive and quantitative data directly available in the articles (e.g., study design, sample size, VR system) were compared verbatim. However, approximations were accepted for qualitative outcomes (e.g., barriers, facilitators) and outcomes requiring higher-order reasoning not necessarily stated directly in the article (e.g., setting, intervention intensity). Instances of qualitative approximations for descriptive or quantitative data fields were marked as incorrect (misses) for accuracy calculations and noted in the researcher's observations. Data points correctly extracted by human researchers and the GPT-5 mode variant were marked as hits and instances where researchers and LLM outputs indicated data were not available/reported were marked as correct rejections. Data captured by the human researchers but not extracted by the LLM (indexed by N/A), incorrectly represented (i.e., misinterpretation), or qualitative approximations (rather than raw data) were marked as misses. Alternatively, if a data point was correctly provided by a GPT-5 mode variant (verified by the first author confirming with the article full-text) that was not captured by the research team (i.e., researchers indexed as N/A but data was available) it was marked as a false alarm.

Article Summarisation

Because additional metrics were specified to inform similarities and differences in article summarisation between LLM and human-generated workflows, signal detection indices focused on comparing the presence/absence of generated summaries between the research team and LLM mode variants. To this end, if a summary was created by a human researcher and the GPT-5 mode variant, and correctly depicted the article content, it was marked as a hit; summaries depicting incorrect content or misattributing information from other articles/sources were marked as misses, along with article summaries that were not generated by the LLM. If a GPT-5 mode variant

produced an article summary for an article not specified in the prompt instructions, this would be marked as a false alarm. Here, it was not possible to quantify correct rejections (summaries not generated by the research team or mode variants).

Critical Appraisals

The GRADE tables generated by GPT-5 mode variants were compared rating-by-rating (n = 8 per study) to the study-specific tables prepared by the research team (with consensus agreement). Each table specified the initial certainty, seriousness ratings for each GRADE criterion (risk of bias, inconsistency, indirectness, imprecision, publication bias), upgrading factors, and overall certainty of the study, with justification notes for each component (see Supplementary Materials #). For quantitative considerations, seriousness ratings were compared with binary criteria (not serious vs. serious/very serious). If human and GPT-5 certainty ratings matched or GRADE components were (binary) classified as serious by humans and the LLM variant, items were marked as hits. In contrast, GRADE components classified as not serious by the research team and GPT-5 mode variant were marked as correct rejections. When GPT-5 outputs were inconsistent with human ratings (considered incorrect) or the LLM did not provide a rating (N/A), items were marked as misses. In cases where a GPT-5 mode variant promoted higher certainty or classified a GRADE component as serious that was rated as not serious by the research team, items were marked as false alarms.

Results Synthesis

The descriptive frequencies and statistics (e.g., mean, median) reported in GPT-5 mode variant outputs were compared item-wise with values derived by the research team. Correct values identified in human- and GPT-generated outputs were marked as hits, whereas values incorrectly produced or not calculated (N/A) by the LLM were marked as misses. Here, false alarms were

comprised of values generated by a GPT-5 model variant that were marked as N/A by researchers. Correct rejections were not possible to quantify in this case (i.e., values not calculated by the research team or LLM variants).

The first author reviewed the human-generated narrative observations to identify the main messages conveyed by the research team for feasibility (e.g., acceptability, suitability for depression), intervention components (e.g., gamification features, delivery), and treatment potential/efficacy (e.g., improvements in depression symptoms). Overall, five key points were identified for feasibility, three for intervention components, and four key points relevant to treatment potential/efficacy. The first author then reviewed each GPT-5 mode variant output, highlighting the main messages identified by the LLM. If a pivotal concept was mentioned in human researchers' qualitative observations and the GPT-5 mode variant's narrative synthesis, it was marked as a hit. If a message was emphasized in the human narrative that was not captured in the LLM synthesis, it was marked as a miss. However, messages that were highlighted in a GPT-5 output but not captured by the research team were marked as false alarms. In this case, false alarms were not necessarily erroneous and could reflect novel insights overlooked by the human researchers.
