# Supplementary material for: Leveraging prompt-driven generative AI for systematic reviews in digital psychiatry: A stage-matched comparative proof-of-concept for healthcare researchers and clinicians
Source: PLOS Digit Health. 2026 Sep 10;5(9):e0001666. doi: 10.1371/journal.pdig.0001666 (PMC13561331; doi:10.1371/journal.pdig.0001666)
Supplement: Supplementary file 6 [file pdig.0001666.s006.pdf]

S1 Table: Stage-specific time and personnel requirements across the SLR workflows

Table S6.1 Time and personnel requirements for SLR tasks across the GPT-5 and human-led workflows.

Task	Estimated Time (mins)				Personnel*			
	Auto	*Agent*	*Deep Research*	*Human(s)^*	*Auto*	*Agent*	*Deep Research*	*Human(s)*
Preliminary Searches	0.67	6	14	780	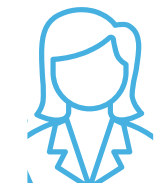	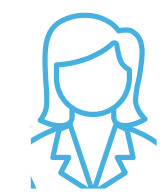	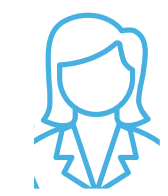	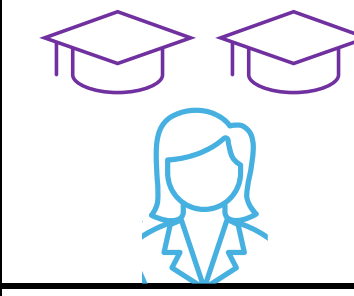
Research Question Development	1	1	12	120	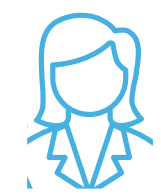	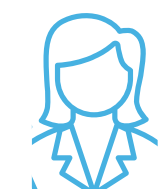	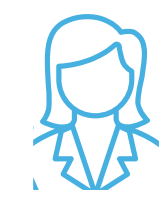	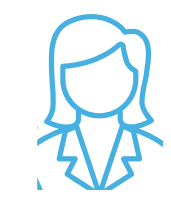
Eligibility Criteria	1	3	8	600	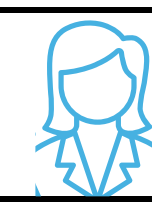	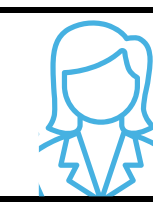	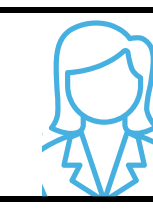	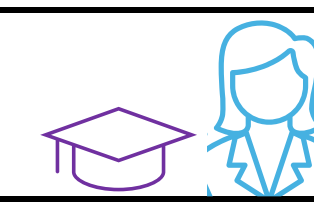
Search Strategy	1	1	11	1320	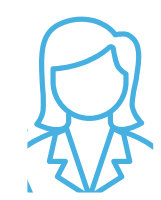	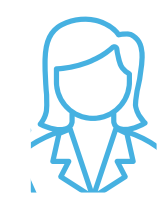	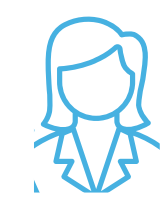	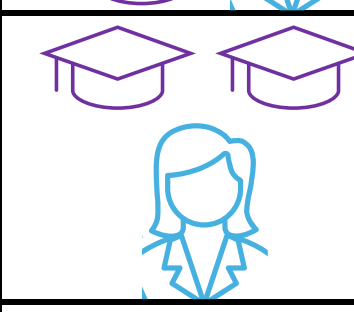
Article Screening	0.5	4	34	3300	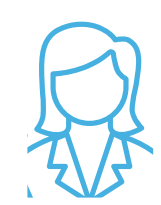	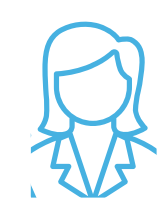	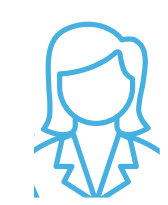	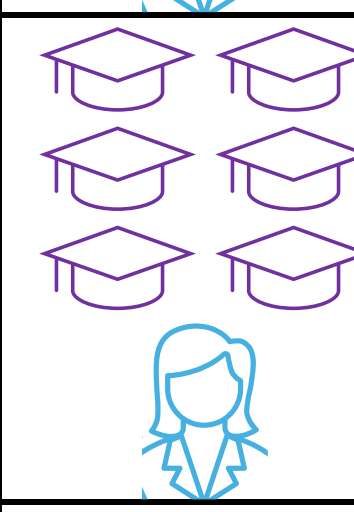
Data Extraction	1	8	77	1200	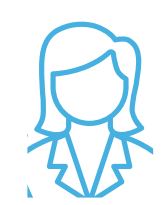	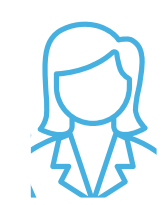	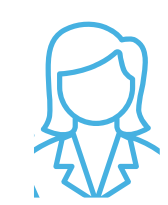	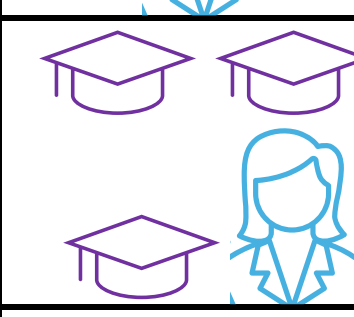
Article Summaries	1	0.77	44	1080	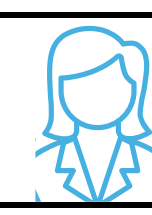	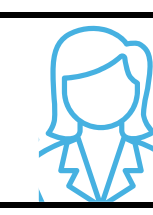	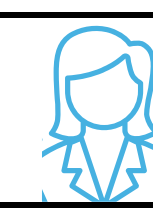	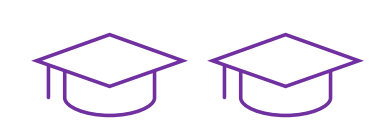

Critical Appraisals	0.72	3	16	1100				
Results Synthesis	0.08	4	24	660				

* Student researchers ( ) had no financial costs. Post-doctoral research fellow ( ) costs are based on a CIHR-funded salary of $65,000 (approximately $33.19 CAD/hour).

^ Cumulative time across all research personnel (student researchers and/or the research fellow).
