# Supplementary material for: Leveraging prompt-driven generative AI for systematic reviews in digital psychiatry: A stage-matched comparative proof-of-concept for healthcare researchers and clinicians
Source: PLOS Digit Health. 2026 Sep 10;5(9):e0001666. doi: 10.1371/journal.pdig.0001666 (PMC13561331; doi:10.1371/journal.pdig.0001666)
Supplement: Supplementary file 7 [file pdig.0001666.s007.pdf]

S1 Template: GRADE critical appraisal table template

Feasibility and treatment potential of immersive virtual reality exergaming for depression: Systematic literature review and meta-analysis

Study 1		
**Domain**	**Assessment (Tick / Rate)**	**Justification / Notes**
**Initial Certainty**	High / Low	
**Risk of Bias**	Not serious (0) / Serious (–1) / Very serious (–2)	
**Inconsistency**	Not serious (0) / Serious (–1) / Very serious (–2)	
**Indirectness**	Not serious (0) / Serious (–1) / Very serious (–2)	
**Imprecision**	Not serious / Serious (–1) / Very serious (–2)	
**Publication Bias**	Not serious / Serious (–1) / Very serious (–2)	
**Upgrading Factors (non-RCT only)**	Large effect (+1 or +2) / Dose-response (+1) / All plausible confounding (+1)	
**Overall Certainty (Final Rating)**	High / Moderate / Low / Very low	
