# Supplementary material for: Leveraging prompt-driven generative AI for systematic reviews in digital psychiatry: A stage-matched comparative proof-of-concept for healthcare researchers and clinicians
Source: PLOS Digit Health. 2026 Sep 10;5(9):e0001666. doi: 10.1371/journal.pdig.0001666 (PMC13561331; doi:10.1371/journal.pdig.0001666)
Supplement: Supplementary file 8 [file pdig.0001666.s008.pdf]

S2 Tables: Signal detection values and bounded reclassification sensitivity analyses across SLR tasks

Table S8.1 Signal detection outcome frequencies (hits, misses, false alarms, correct rejections, and *d′* for GPT-5 mode variants (Auto, Agent, and Deep Research) relative to human researcher outputs, across SLR workflow tasks.

Task	Hits			Misses			False Alarms			Correct Rejections			d′		
	Auto	*Agent*	*Deep Research*	*Auto*	*Agent*	*Deep Research*	*Auto*	*Agent*	*Deep Research*	*Auto*	*Agent*	*Deep Research*	*Auto*	*Agent*	*Deep Research*
Preliminary Searches	1	3	1	34	28	33	0	0	0	0	7	0	-	-	-
Research Question Development	3	5	5	4	2	1	2	4	3	N/A	N/A	N/A	0.24	0.23	0.57
Eligibility Criteria	5	4	4	4	4	4	0	0	0	3	4	4	-	-	-
Search Strategy	10	8	8	24	30	30	5	2	2	N/A	N/A	N/A	-	-	-
Article Screening – Title/Abstract	0	48	14	56	8	42	0	333	0	2840	2520	2894	-	**2.23**	-
Article Screening – Full Text	0	0	9	10	10	1	0	0	1	0	371	4	-	-	**1.77**
Data Extraction	100	274	37	337	208	756	55	31	2	305	285	3	0.28	**1.46**	-1.46
Article Summaries	10	10	8	0	0	2	0	0	0	N/A	N/A	N/A	-	-	-
Critical Appraisals	20	24	N/A	8	27	N/A	17	15	N/A	35	34	N/A	0.98	0.42	-
Results Synthesis	24	58	51	46	9	20	2	9	5	1	9	9	-0.72	**1.08**	0.90

- Value cannot be calculated

Table S8.2 Bounded reclassification sensitivity analysis for SLR screening tasks, assuming the current proof-of-concept consensus-based human reference workflow is in line with ~80% human reference standards for medical SLRs. Lower human error scenarios simulate sensitivity, specificity, and accuracy performances as error levels improve.

Model	Task	Human Error Scenario	Sensitivity	Specificity	Accuracy
Auto	Title/Abstract Screening	20%	0.00	1.00	0.98
Auto	Title/Abstract Screening	10%	0.00	1.00	0.98
Auto	Title/Abstract Screening	0%	0.00	1.00	0.98
Agent	Title/Abstract Screening	20%	0.86	0.88	0.88
Agent	Title/Abstract Screening	10%	0.63	0.87	0.87
Agent	Title/Abstract Screening	0%	0.89	0.86	0.86
Deep Research	Title/Abstract Screening	20%	0.25	1.00	0.99
Deep Research	Title/Abstract Screening	10%	0.23	1.00	0.98
Deep Research	Title/Abstract Screening	0%	0.22	1.00	0.98
Auto	Full Text Screening	20%	0.00	-	0.00
Auto	Full Text Screening	10%	0.00	-	0.00
Auto	Full Text Screening	0%	0.00	-	0.00
Agent	Full Text Screening	20%	0.00	1.00	0.97
Agent	Full Text Screening	10%	0.00	1.00	0.97
Agent	Full Text Screening	0%	0.00	1.00	0.97
Deep Research	Full Text Screening	20%	0.90	0.80	0.87
Deep Research	Full Text Screening	10%	0.89	0.78	0.85
Deep Research	Full Text Screening	0%	0.88	0.77	0.85

- Value cannot be calculated

* Note: Benchmark data from the current proof-of-concept are highlighted in light grey as the reference standard.

Table S8.3 Bounded reclassification sensitivity analysis assuming the current proof-of-concept consensus-based human reference workflow is considered absolute ground truth, with no error. Higher human error scenarios simulate sensitivity, specificity, and accuracy performance as error levels worsen.

Model	Task	Human Error Scenario	Sensitivity	Specificity	Accuracy
Auto	Title/Abstract Screening	0%	0.00	1.00	0.98
Auto	Title/Abstract Screening	10%	0.00	1.00	0.98
Auto	Title/Abstract Screening	20%	0.00	1.00	0.98
Agent	Title/Abstract Screening	0%	0.86	0.88	0.88
Agent	Title/Abstract Screening	10%	0.92	0.89	0.89
Agent	Title/Abstract Screening	20%	0.95	0.90	0.91
Deep Research	Title/Abstract Screening	0%	0.25	1.00	0.99
Deep Research	Title/Abstract Screening	10%	0.27	1.00	0.99
Deep Research	Title/Abstract Screening	20%	0.30	1.00	0.99
Auto	Full Text Screening	0%	0.00	-	0.00
Auto	Full Text Screening	10%	0.00	-	0.00
Auto	Full Text Screening	20%	0.01	-	0.01
Agent	Full Text Screening	0%	0.00	1.00	0.97
Agent	Full Text Screening	10%	0.00	1.00	0.98
Agent	Full Text Screening	20%	0.01	1.00	0.98
Deep Research	Full Text Screening	0%	0.90	0.80	0.87
Deep Research	Full Text Screening	10%	0.91	0.82	0.88
Deep Research	Full Text Screening	20%	0.92	0.83	0.89

- Value cannot be calculated

* Note: Benchmark data from the current proof-of-concept are highlighted in light grey as the reference standard.
